# Computational Intelligence and Wavelet Transform Based Metamodel for Efficient Generation of Not-Yet Simulated Waveforms

**DOI:** 10.1371/journal.pone.0146602

**Published:** 2016-01-08

**Authors:** Gabriel Oltean, Laura-Nicoleta Ivanciu

**Affiliations:** Department of Bases of Electronics, Technical University of Cluj–Napoca, Romania; University of California Berkeley, UNITED STATES

## Abstract

The design and verification of complex electronic systems, especially the analog and mixed-signal ones, prove to be extremely time consuming tasks, if only circuit-level simulations are involved. A significant amount of time can be saved if a cost effective solution is used for the extensive analysis of the system, under all conceivable conditions. This paper proposes a data-driven method to build fast to evaluate, but also accurate metamodels capable of generating not-yet simulated waveforms as a function of different combinations of the parameters of the system. The necessary data are obtained by early-stage simulation of an electronic control system from the automotive industry. The metamodel development is based on three key elements: a wavelet transform for waveform characterization, a genetic algorithm optimization to detect the optimal wavelet transform and to identify the most relevant decomposition coefficients, and an artificial neuronal network to derive the relevant coefficients of the wavelet transform for any new parameters combination. The resulted metamodels for three different waveform families are fully reliable. They satisfy the required key points: high accuracy (a maximum mean squared error of 7.1x10^-5^ for the unity-based normalized waveforms), efficiency (fully affordable computational effort for metamodel build-up: maximum 18 minutes on a general purpose computer), and simplicity (less than 1 second for running the metamodel, the user only provides the parameters combination). The metamodels can be used for very efficient generation of new waveforms, for any possible combination of dependent parameters, offering the possibility to explore the entire design space. A wide range of possibilities becomes achievable for the user, such as: all design corners can be analyzed, possible worst-case situations can be investigated, extreme values of waveforms can be discovered, sensitivity analyses can be performed (the influence of each parameter on the output waveform).

## Introduction

The standard circuit-level simulation based design approach can only be used when a designer has sufficient time for running simulations to optimize the circuit. Unfortunately, this is usually not the case, since the timeline for a design process is very short. Simulation times for complex circuits are, most commonly, very long and it is not feasible to conduct an exhaustive search to find the optimal circuit [1]. The use of a model that provides satisfactory simulation results, while keeping the simulation time at acceptable values, comes as a natural course of action.

That particular model cannot be the circuit model, because it would lead to long and complex simulations, but something with a higher level of abstraction; moreover, that particular model should be something that doesn’t actually simulate the circuits, but only produces their output waveforms. Hence, the idea of a metamodel, or a model of the model, emerges. Generally speaking, a metamodel, or a surrogate model, is a model of the model, i.e. a simplified model of an actual model of a circuit, system, or software like entity [2–3]. A metamodel can be a mathematical relation or algorithm representing input and output relations. A model is an abstraction of phenomena in the real world; a metamodel is yet another abstraction, highlighting properties of the model itself. Various types of metamodels include polynomial equations, neural network, Kriging, etc. [2]. Metamodeling typically involves studying the input-output relationships and then fitting proper metamodels to represent that behavior.

Once the metamodels are generated, the designer can conduct more extensive analyses of the circuit and use the same metamodel for different criteria to be optimized. The detailed simulation is significantly more time consuming than using the metamodel. The designer can adjust and change the optimization algorithm to fit the proposed design flow, especially if the circuit undergoes multiple optimization (automatic) iterations [1].

The metamodeling process implies a mathematical representation of the output, based on a prediction equation or algorithm, language and tool independent, reusable for different specifications, and which can be applied using non-EAD tools like MATLAB [2]. The key points of metamodeling, according with [2] are:

- accuracy—capability of generating the system response over the design space;
- efficiency—computational effort required for constructing the metamodel;
- transparency—capability of providing the information concerning contributions and variations of design variables and correlation among the variables;
- simplicity—simple methods should require less user input and be easily adapted to different problem.

Computational intelligence is an umbrella concept that includes practical adaptation and self-organization concepts, and also algorithms and implementations that facilitate an intelligent behavior, in a complex and variable environment [4–5]. Computational intelligence is successfully applied in solving incompletely described problems or problems that are described using formal models, implying highly consuming algorithms, which makes it a perfect candidate for the use in the development of metamodels [6–7].

According with our knowledge, there are not systematic approaches of generating complete waveforms in different points of a complex system as a function of input parameter combinations, using a cheap and fast substitute (e.g. metamodeles) for extensive simulation. However, a literature review shows that efforts have been put into the approximation of waveforms, regardless of their nature (electrical or non-electrical), using various techniques.There are some methods [8–9] which apply heuristics to the outputs of the simulation, in order to predict only not-yet simulated points, not the full waveform. A series of approaches, such as [10], imply applying statistics on the output values, but do not build predictive models of the signal of interest, therefore can only assess the quality of the system under consideration, not to improve it. Our metamodel is more generous addressing the generation of the entire waveform at once, and thus giving the designer the full perspective of the system behaviour.

A method to predict post-layout waveform by System Identification, based on the fact that the waveforms of pre-layout and post-layout are always correlated, is described in [11]. It uses linear models (linear ARX, impulse response, and transfer function) and non-linear models (non-linear ARX and Hammerstein-Wiener). The predicted waveforms cannot always achieve very high accuracy; however, they can essentially predict the trends of the waveforms, which can guide the designers to diagnose and optimize their designs. Our metamodel provides high approximation accuracy, making it a reliable starting point for designers, in the process of design diagnosis and optimization.

A solution to the problem of quickly and accurately predicting gravitational waveforms within any given physical model is discussed in [12]. The solution constructs a surrogate model for a fiducial set of waveforms in three offline steps. In the first step, a reduced basis is generated that spans the space of waveforms in the given range of parameters. In the second step, an application-specific (i.e., empirical) interpolant is constructed using only the reduced basis waveforms. In the third step, a fit for the parametric dependence of the waveform’s phase and amplitude at each empirical time is implemented. Results show that these surrogate models provide a reduction of the evaluation time with three orders of magnitude, compared to the standard methods, while maintaining a high accuracy. The metamodels proposed in our paper are also developed offline, and require very short evaluation times.

On the other hand, there are some approaches of using ANNs to tackle different issues in waveform processing. Back Propagation Neural Network and Radial Basis Function Neural Network are used to develop behavioural models of a RF power amplifier, the predicted output signal corresponding to sampling points of the amplifier output waveform value [13]. In [14], an ANN is used for detection and classification of electrical disturbances in three-phase systems. Automatic detection of spikes in electroencephalograms (EEG) can be solved using neural networks, as described in [15].

In [16], a neural network provides a means of determining a degree of belief for each identified disturbance waveform in Power System. Three types of neural networks (multilayer perceptron, radial basis function and wavenet) are used in [17] to estimate the feedback signal for a vector controlled induction motor drive.

In [18] the configuration of an ANN (number of hidden units in the hidden layer, transfer function to use at the hidden layer, and transfer function to use at the output layer) is optimized such that the network’s approximation error for signal approximation problems is minimized. Three different signals were considered there: Boolean XOR function, sinusoidal signal, and a signal representing the activity measurements on a server system.

[19] proposes a method that combines ANNs with the Wavelet Decomposition (WD) to generate short-term global horizontal solar radiation forecasting, which is an essential information for evaluating the electrical power generated from the conversion of solar energy into electrical energy. The forecasts derived from the proposed method had a significantly higher correlation with the time series observations of global horizontal solar radiation when compared with the forecasts arising from using only the ANN (i.e., without considering the wavelet signals as input patterns).

Global or local thresholds can be used to separate the most important coefficients of the wavelet decomposition, which can further on be shank [20] or normalized. Such a technique is applied in [21] for noise reduction in cochlear implants. An energy-based wavelet denoising method is proposed in [22], and applied to hydrological time series.

The novel contribution of this paper is the methodology to build metamodels for generating not-yet simulated waveforms of complex systems. The resulted metamodels are very accurate and fast to evaluate, offering new possibilities to the designers to efficiently analyze the full design space, in the quest for optimal design solutions. Specifically, the contributions of this paper are:

1. an efficient method for data-driven metamodel development;
2. integration of several computational techniques in one product, to valorize their advantages (GA for robust optimization, fuzzy sets to formulate optimization objectives, and ANN to learn complex, multidimensional relationships between a parameter set and a set of coefficients);
3. utilization of multilevel wavelet decomposition to extract the most representative features of waveforms and multilevel wavelet reconstruction to generate highly reliable approximations of new waveforms;
4. a case study for three waveform families corresponding to a complex system (10 dependent parameters) for safety in the automotive industry;
5. a final discussion, that includes performance analysis, necessary resources for metamodel development and utilization, and perspectives for the metamodels’ utility and further development.

The paper is structured as follows: Section 2 “Overview of the proposed metamodel” briefly describes the overall structure of the metamodel, Section 3 “Metamodel development” presents the three steps that were used in order to build the metamodel: GA optimization, training data set generation and building the ANN. The presentation and analysis of the method implementation and results, for each of the three waveform families are addressed in Section 4 “Implementation and experimental results”. Section 5 “Discussion” synthesizes the performance of the metamodels, and also gives possible usage alternatives, while Section 6 “Conclusions” summarizes the paper.

## Overview of the Proposed Metamodel

The system considered here as a case study is a typical ECU (electronic control unit) for which the control signal is influenced both by the DUT (device under test) as well as the load variation, as described in [23]. The exact switch-on time, given as value and pulse duration, is crucial when it comes to driving the squib of the airbag. The SystemC-AMS model is subject to simulations, to extract the output signals corresponding to applied variations on the DUT and Load parameters (Table II in [23]. A number of 10 different parameters that can influence the system operation are considered. For each parameter, a nominal, a maximum and a minimum value was defined.

Signals in different points of the system are generated by simulation for the case when all parameters take their nominal value, obtaining the nominal waveform for each signal. To generate more data, meaning more waveforms for each signal, the system under consideration was simulated for another 200 different combinations of parameters. As a result, for each signal, there is a family of 200 different waveforms, plus the nominal waveform. In the present work, three families of waveform (1^st^ family, 2^nd^ family and 3^rd^ family) are considered. These waveform families are described in more detail in section “Implementation and experimental results.

### Structure of the metamodel

**Fig 1:**
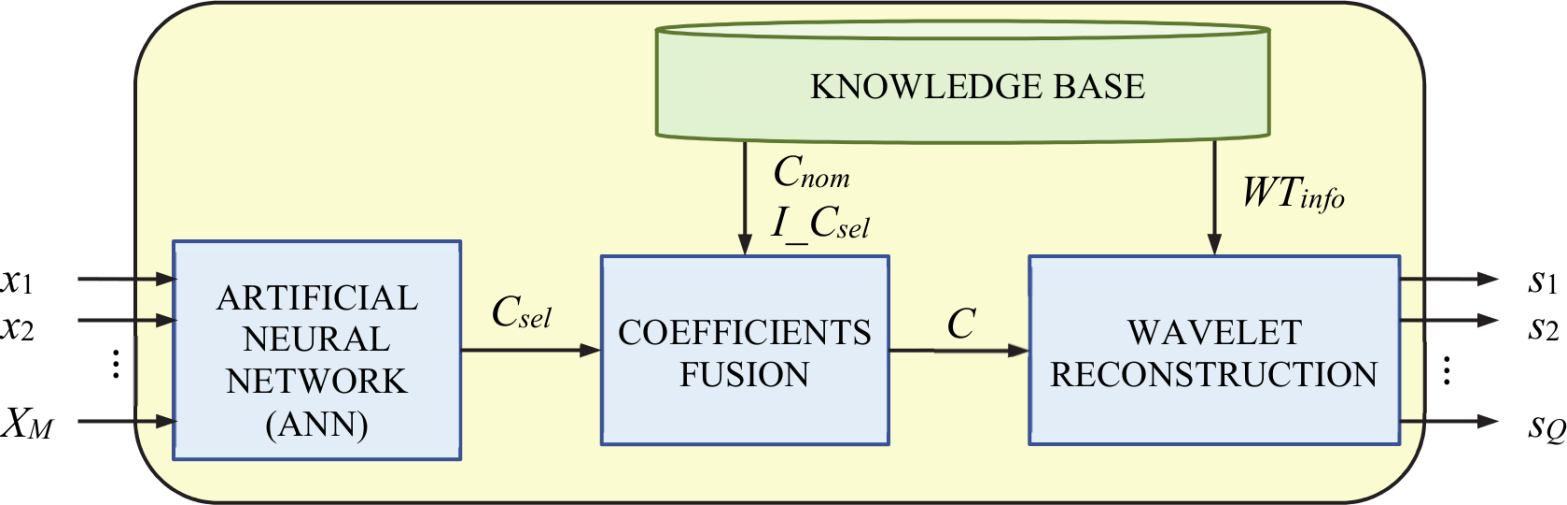
Block diagram of the proposed metamodel for waveform generation.

The block diagram of our proposed metamodel is presented in Fig 1.

The proposed metamodel for waveform generation has to rapidly produce an accurate approximation of the output waveform by its time samples *S* = {*s*_1_, *s*_2_, …, *s*_*Q*_} (*Q* is the number of samples) for any combination of the input parameters *X* = {*x*_1_, *x*_2_, …, *x*_*M*_} (*M* is the total number of parameters).

The proposed metamodel is a knowledge-based metamodel, containing one block that stores knowledge (KNOWLEDGE BASE) and three computation blocks (ARTIFICIAL NEURAL NETWORK, COEFFICIENTS FUSION, and WAVELET RECONSTRUCTION). The metamodel operation is based on the principle of generating all time samples of the output waveform using a wavelet reconstruction transform. To do this, it is necessary to use information about the wavelet transform, *WT*_*info*_ (mother wavelet and bookkeeping vector) and the vector of the coefficients, *C*. The vector of coefficients *C* is provided by the COEFFICIENTS FUSION block, through the fusion between the vector of nominal coefficients, *C*_*nom*_ and the vector of selected coefficients, *C*_*sel*_. The fusion consists in replacing every nominal coefficient by its corresponding one, from the vector of selected coefficients *C*_*sel*_, as indicated by the vector of indices of selected coefficients, *I*_*C*_*sel*_. *C*_*nom*_ is the vector of coefficients resulted from the wavelet decomposition of the nominal waveform, according with *WT*_*info*._ *I*_*C*_*sel*_ is the vector of the indices of the most relevant coefficients from an optimal wavelet decomposition, as it will be explained later, in the “GA optimization” section. The information about the wavelet transform, *WT*_*info*_, the vector of nominal coefficients, *C*_*nom*_, and the vector of indices of selected coefficients, *I*_*C*_*sel*_, are previously determined and stored in the KNOWLEDGE BASE, in the phase of metamodel development, as presented hereinafter.

The vector of selected coefficients, *C*_*sel*_, is computed by the ARTIFICIAL NEURAL NETWORK (ANN), for each combination of input parameters *X*.

### Metamodel Development

The metamodel is developed around the ANN that has the great responsibility to accurately generate its output (selected coefficients) for each combination of input parameters. Developing a fitted ANN requires a supervised learning procedure based on a set of numerical data. The quality of the trained ANN is directly connected with the quality of the data set involved in the training process.

Some of the main features that recommend the artificial neural networks are [24–26]: ANNs are universal approximators that can learn data by example and can approximate any complex nonlinear multi-variable function with any desired accuracy

In our approach, we have used a feed-forward, back-propagation ANN, namely a multi-layer perceptron, with three layers: input, hidden and output. The number of neurons on the input layer is the number of input parameters, while the number of neurons on the output layer is equal to the number of selected coefficients. For the hidden layer, the number of neurons was determined via a series of trial runs, in order to obtain an optimal network structure, with minimum error. A pure linear activation function is used for the output neurons, and a sigmoidal one, for the neurons of the hidden layer.

For the training procedure, the full data set is arbitrarily split into three data subsets: training subset, validation subset and testing subset. The training subset is directly used to train the neural network, by adapting the neural network parameters (weights of the connections between neurons and neuron biases) in each training epoch. The validation subset supervises the training, detecting a possible overfitting phenomenon. Finally, the testing subset measures the performance of the neural network, inasmuch as that it is not at all involved in the training process. When the training phase ends, the user analyses the performances of the trained ANN: evolution and final values of the mean squared error in all three data subsets, error histogram, and linear regression.

If the ANN performances are satisfactory, the process ends and the accepted ANN will be further used in the metamodel. On the contrary, if the performances are not satisfactory, it can be improved by retraining the ANN with the same architecture, but using different initial values for the neural network parameters and a different allocation of data in the three subsets. If the results are still not acceptable, a new architecture of the neural network can be investigated. Further information about ANN generation, training and performance evaluation can be found in [6–7].

To capture all the characteristics of a certain family of waveforms, common and differentiating, in a first approach, it seems that all the time samples should be considered. If the neural network has to generate all those values, this would lead to an ANN with a lot of neurons in its output layer, which is not practical, from the point of view of network training resources (time, computer memory, size of the training data set) and also form the point of view of implementing and simulating such a huge network. To substantially decrease the dimensionality of the problem (far too many neurons in the output layer) we focus on a solution inspired from the pattern recognition domain, where to recognize (classify) a pattern we have to first describe it by a reduced set of representative features [27–28].

The chosen solution is to use a wavelet transform, and to select only the most relevant coefficients from the resulted decomposition, so that the original waveform can be accurately reconstructed, using a wavelet reconstruction.

The wavelet transform is a tool that cuts waveforms into different frequency components, and then studies each component with a resolution matched to its scale [29].The wavelet transform preserves both time and frequency information, in its coefficients. On each level of decomposition, the signal is decomposed into low frequency coefficients (approximation) and high frequencies coefficients (details). The approximations are the high-scale, low-frequency components of the signal while the details are the low-scale, high-frequency components [30].

In discrete multilevel wavelet decomposition (DMWD) the decomposition process can be iterated, with successive approximations being decomposed in turn, so that one signal is broken down into many lower resolution components [29–30]. The decomposition can proceed until the individual details consist of a single sample. In practice, a suitable number of levels based on the nature of the signal will be selected. Given a signal of length *K*, the DMWD consist of log_2_ *K* levels at most. The first step produces two sets of coefficients: approximation coefficients *A*_1_ and details coefficients *D*_1_. These coefficients are obtained by convolving the signal with a low-pass filter for approximation and with a high-pass filter for details, followed by dyadic decimation (downsampling). The next step splits the approximation coefficients in two parts, using the same scheme, replacing the original signal by *A*_1_, and producing the coefficients on the next level *A*_2_ and *D*_2_, and so on [30–31].

The number of coefficients on the first decomposition level is given by the relation [31]:

$$N_{A_{1}}=N_{D_{1}}=floor\;\left(\frac{K-1}{2}\right)+F$$ (1)

where *K* is the length of the signal (in our case the number of time samples of the waveforms) and 2*F* is the length of each filter (in the case of Daubechies family *F* is the order of mother wavelet).

Further, the number of coefficients for the *j*^*th*^ decomposition level can be determined with the relation:

$$N_{A_{j}}=N_{D_{j}}=floor\;\left(\frac{N_{A_{j-1}}-1}{2}\right)+F$$ (2)

The mathematical apparatus and further details regarding the wavelet transform can be found in [29],[32].

In this work we have used DMWD, three wavelet families (Daubechies, Symlets and Coiflets), and *L =* 10 as the maximum decomposition level.

### GA optimization

The problem to be solved here involves two different aspects:

- find an optimum multilevel wavelet decomposition, in terms of mother wavelet and decomposition level;
- select a reduced number of relevant decomposition coefficients.

The above two aspects cannot be separately treated, but unitedly, because the number and position of the selected coefficients are directly connected with the mother wavelet and decomposition level. As a consequence, an optimization method is required to quest for the best combination of mother wavelet, decomposition level and selected coefficients. Our optimization problem has conflicting objectives: on one hand we need high accuracy, meaning the most appropriate wavelet transform for the waveform family, keeping as many decomposition coefficients as possible; on the other hand, we want dimensionality reduction (small complexity), meaning as little selected coefficients as possible.

To find an at least good enough solution (not always necessarily the best one) to the above presented optimization problem, we are using a genetic algorithm.

Genetic algorithms are a particular class of evolutionary algorithms, that use biology inspired techniques such as selection, mutation, crossover and inheritance. In order to explore the entire solution space, GAs use populations of solutions, which evolve with each generation. GA techniques have been shown to efficiently search large solution spaces, containing discrete or discontinuous parameters and non-linear constraints, without being trapped in local minima [33]. Detailed information about how genetic algorithms work can be found in [6–7].

To maintain the population diversity and to avoid a fast polarization towards one multidimensional point in the solution space, the population is distributed into two sub-populations. The number of individuals will be determined after a series of trial-and-error runs, taking into account the computational resources and performance.

The individuals evolve with every iteration, by means of the genetic operators. First, a rank fitness scaling, followed by roulette selection is applied, in order to select the individuals that will contribute to the development of the new sub-populations. The best 2 individuals (elite count) will automatically be sent to the new population, without any changes. Intermediate crossover, with a crossover fraction of 0.7, and mutation are applied to the remaining selected individuals in each sub-population. Every 6 epochs (migration interval), individual migrate from one sub-population to the other. The algorithm stops when the average relative change in the fitness function value over 7 generations (stall generations) is insignificant.

**Fig 2:**
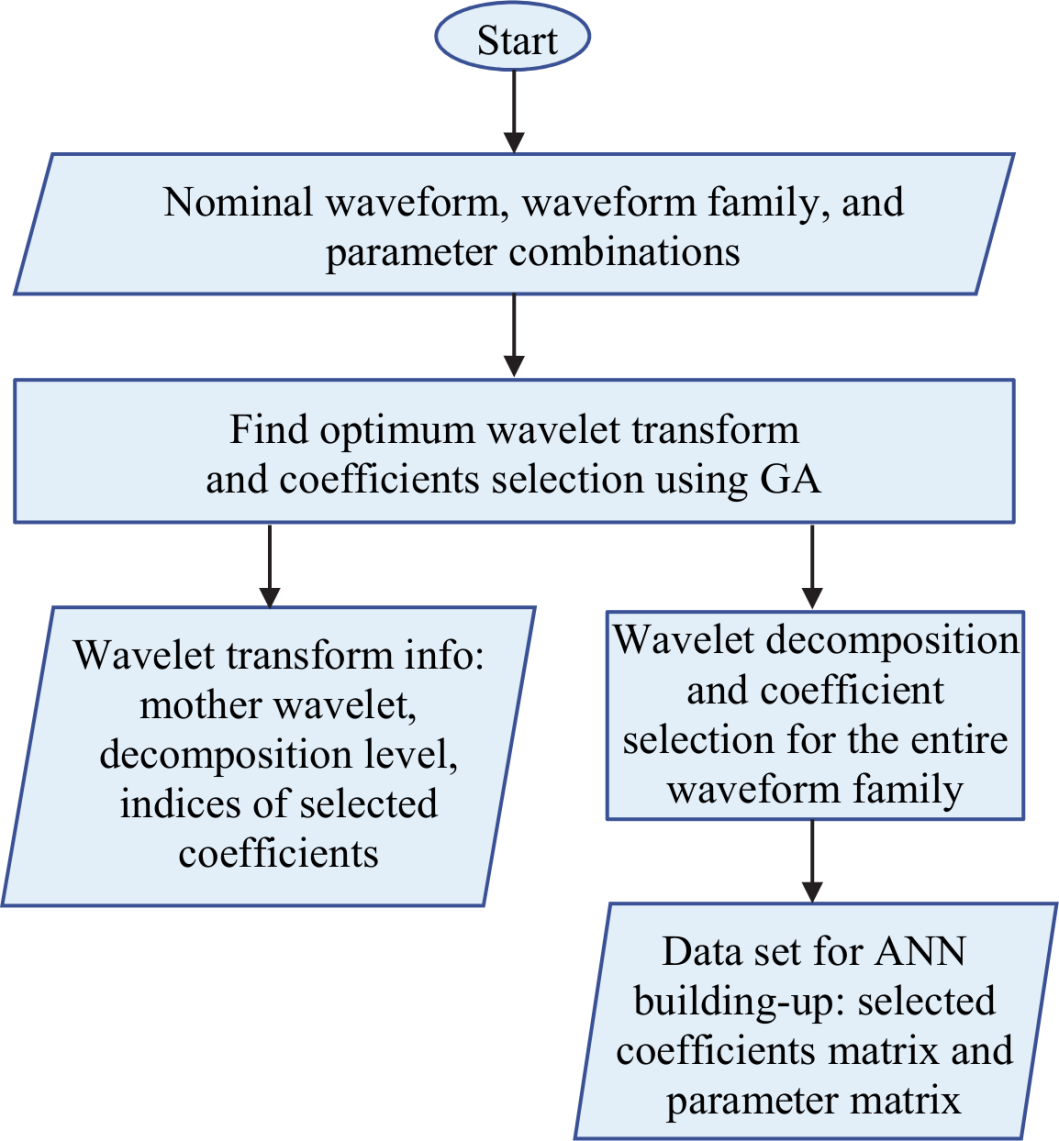
The GA optimization process to obtain the optimum wavelet decomposition, selected coefficients, and training data set.

The GA optimization process is used to find the most appropriate wavelet transform, select the relevant coefficients, and eventually obtain the data set for ANN building-up. The optimization process, presented in Fig 2, starts from the available data: nominal waveform (corresponding to the nominal value of the parameters), waveform family (waveforms corresponding to different combinations of the parameter), and parameter combinations.

Next, we enter the optimization phase, to discover an optimum solution for the wavelet decomposition and selection of the coefficients. The chromosome consists of three genes:

[*wavelet_name*; *decomposition_level*; *selected_coefficients_ratio*]

The optimization problem is a multiobjective optimization one, composed by two objectives: high accuracy approximation of the wavelet decomposition and reduced number of selected coefficients. The accuracy of each approximation of the wavelet decomposition is evaluated by means of the mean squared error (*mse*) between the original and reconstructed waveforms:

$$mse=\frac{1}{K-1}\sum_{k=1}^{K}{\left(s_{k}^{r}-s_{k}^{o}\right)}^{2}$$ (3)

where *K* is the number of time samples, $s_{k}^{r}$ is the *k*^*th*^ reconstructed time sample, and $s_{k}^{o}$ is the *k*^*th*^ original time sample. The reconstructed waveform is obtained using the same wavelet transform as for the decomposition, but taking only the selected coefficients from the original wavelet decomposition, the rest of the coefficients being the ones taken from the decomposition of the nominal waveform.

Across the entire waveform family we sum up all the *mse*s for each individual waveform, to obtain a global accuracy estimation, *gmse*, as defined in Eq 4:

$$gmse=\sum_{j=1}^{N}\left(\frac{1}{K-1}\sum_{k=1}^{K}{\left(s_{kj}^{r}-s_{kj}^{o}\right)}^{2}\right)$$ (4)

where *N* is the total number of waveforms, *K* is the number of time samples, $s_{kj}^{r}$ and $s_{kj}^{o}$ are the *k*^*th*^ reconstructed, respectively original time samples for the *j*^*th*^ waveform in the family.

To appreciate the number of selected coefficients, we are using the selected coefficients ratio (*scr*), as the ratio between the number of selected coefficients and the number of time samples:

$$\mathrm{scr}=\frac{\mathrm{number of selected coefficients}}{\mathrm{number of time samples}}$$ (5)

To select the coefficients, a global threshold across all decomposition levels and across the entire waveform family is used. For the *j*^*th*^ waveform in the family, the full vector of decomposition coefficients *C*_*j*_ is:

$$C_{j}=[c_{j1},c_{j2},\ldots ,c_{jl},\ldots ,c_{jL}]$$ (6)

where *c*_*jl*_ is the *l*^*th*^ coefficient in the decomposition and *L* is the number of coefficients. We compute the vector of accumulated decomposition coefficients, *AC* by summing up the absolute values of the individual coefficients at every *l* location:

$$AC=[ac_{1},ac_{2},\ldots ,ac_{l},\ldots ,ac_{L}]$$ (7)

$$ac_{l}=\sum_{j=1}^{N}\left|c_{jl}\right|$$ (8)

The selected coefficients are the ones corresponding to the position where the value of the accumulated coefficient is greater than or equal to the global threshold. In order to select a number of coefficients in accordance with the *scr* value obtained from the GA, the global threshold is dynamically determined for each individual in the population.

**Fig 3:**
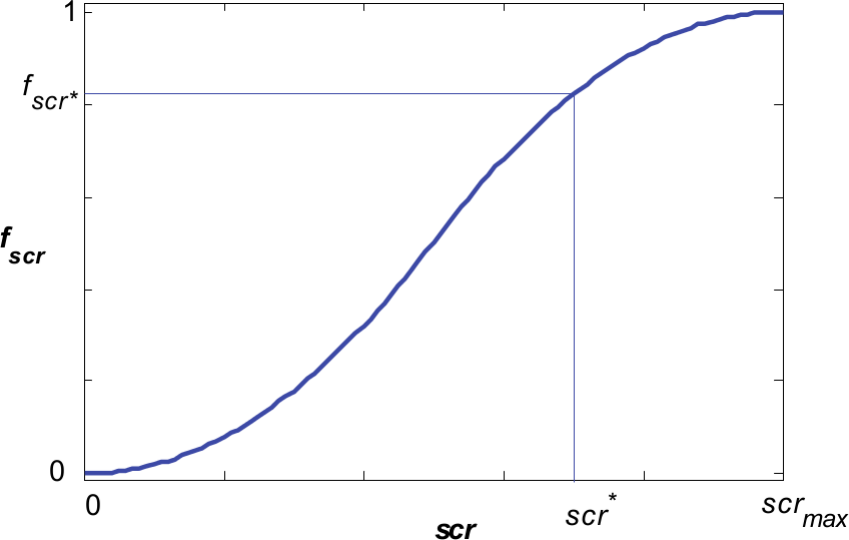
Defining the objective function for *scr* using an *s*-type fuzzy set.

This multiobjective optimization problem is then transformed into a single-objective optimization one, by combining the two metrics, *gmse* and *scr*, into one objective function. The dynamic ranges for these metrics are very different, so, in order to make them comparable during the genetic evolution, we first use a domain transformation, by means of an *s*-type fuzzy set, followed by a weighted summation. Fig 3 presents the *s-*type fuzzy set used for the domain transformation of *scr*. It makes the transformation from the [0, *scr*_*max*_] domain into the [0, 1] domain:

$$f_{scr}\;:\;[0,\;scr_{\text{max}}]\to [0,\;1]$$ (9)

where *scr*_*max*_ is the upper bound of *scr*, to be set by the user.

As an illustration, if the current value for *scr* is scr^*^, the value of the objective function results as $f_{scr*}$.

A similar function, *f*_*gmse*_, is used to define the objective function associated with *gmse*:

$$f_{gmse}\;:\;[0,\;gmse_{\text{max}}]\to [0,\;1]$$ (10)

where *gmse*_*max*_ is the upper bound of *gmse*.

Now, both individual objective functions share the same dynamic range, [0, 1]. Using a weighted sum, the final scalar objective function *f* results as:

$$f=w_{1}f_{gmse}+w_{2}f_{scr}$$ (11)

where *w*_1_ and *w*_2_ are the weights or relative preferences associated with the individual objective functions.

Running the GA optimization produces: the wavelet name, the decomposition level (bookkeeping vector) and the vector containing the indices (locations) of the selected coefficients. This information is stored into the knowledge base of the metamodel (*WT*_*info*_ and *I_C*_*sel*_*−*see Fig 1), and also used for generation of the necessary data training set.

### Training data set generation

Each waveform in the family is decomposed using the optimized wavelet transform. From the resulted decomposition coefficients, a coefficients selection operation takes place, according with the indices of selected coefficients. The selected coefficients for all waveforms in the family are then organized into the selected coefficient matrix, *SC*, to be used as output data in the training data set:

$$SC=\left[\begin{array}{cccc} sc_{11} & sc_{12} & \cdots & sc_{1N}\\ sc_{21} & sc_{22} & \cdots & sc_{2N}\\ \vdots & \vdots & \vdots & \vdots \\ sc_{Q1} & sc_{Q2} & \cdots & sc_{QN} \end{array}\right]$$ (12)

In the *SC* matrix, each column represents the vector of selected coefficients for one waveform, *N* is the number of waveforms and Q is the number of selected coefficients.

The input data in the training data set consists in the parameter combinations, organized in matrix *P*:

$$P=\left[\begin{array}{cccc} p_{11} & p_{12} & \cdots & p_{1N}\\ p_{21} & p_{22} & \cdots & p_{2N}\\ \vdots & \vdots & \vdots & \vdots \\ p_{M1} & p_{M2} & \cdots & p_{MN} \end{array}\right]$$ (13)

The number of rows (*M*) represents the number of input parameters that are varied, while the number of columns is given by the number of combinations of input parameters *N*, which is, in fact, the number of available waveforms in the family.

### Building the ANN

**Fig 4:**
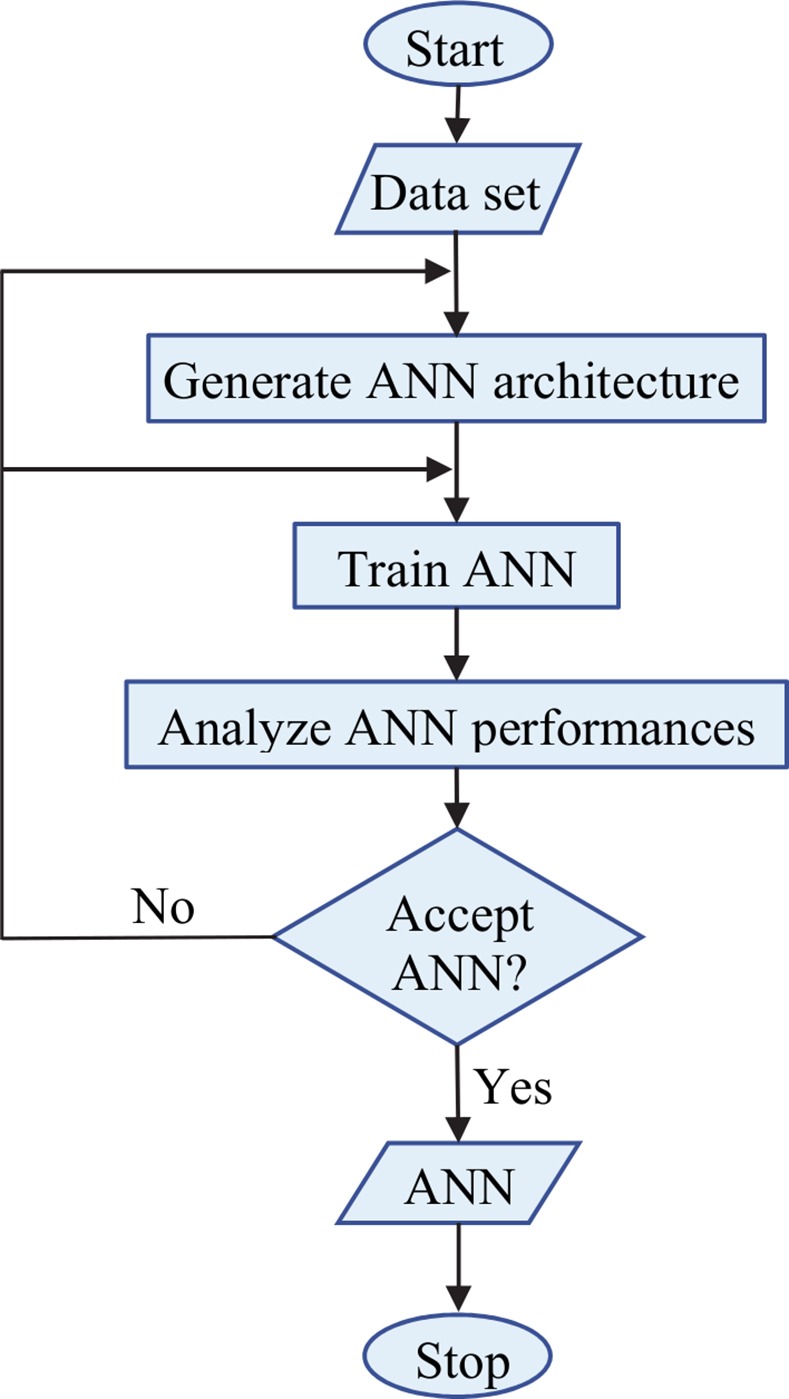
The diagram of building-up the ANN.

The diagram illustrating the ANN build-up process is depicted in Fig 4. Developing the fitted ANN requires a supervised learning procedure based on a numerical data set. The necessary data set is the one determined in the previous paragraph, the matrix *P* representing the input data, while the matrix *SC* represents the output data.

The architecture of the ANN is first decided upon: a multi-layer perceptron with three layers, input, hidden and output. According with [34], where it is stated that, from a function approximation perspective, the single hidden layer is quite adequate as the basic topology and due to the fact that a two-layer feed-forward network (one hidden layer and one output layer) with sigmoid hidden neurons and linear output neurons, can fit multi-dimensional mapping problems arbitrarily well [35], this is the solution for the neural network architecture adopted here.

The number of neurons on each layer is chosen as previously described, at the beginning of the current section. The ANN is then trained until its performance is considered acceptable. The trained ANN is now ready to be used in the metamodel. Should the designer find the performance of the trained ANN not satisfactory, the entire build-up process can be re-run.

## Implementation and Experimental Results

The implementation of the proposed metamodel was carried out in the MATLAB integrated development environment, making good use of the built-in functions available in the Genetic Algorithms, Neural Networks and Fuzzy Logic Toolboxes; a series of custom functions and scripts were also necessary, especially for the metamodel development part of the process, but also for graphical illustration purposes.

The data used for conducting the implementation and experimentation for the above proposed solution consists of some waveform families, as discussed in the first part of the section “Overview of the proposed metamodel”. Let us recall that there are three family of waveforms, each of them composed by a set of 200 waveforms (*N* = 200), simulated for 200 different combinations of 10 input parameters (*M* = 10) and a nominal waveform. Every waveform is described by a number of 8000 time samples.

We will present the metamodel implementation and experimental results for all these three waveform families, one at a time. The results obtained for the 1^st^ family will be discussed in detail, while for the 2^nd^ and 3^rd^ families, only the most relevant results will be presented.

It is worth to mention that the dynamic ranges for the waveform families are different, so, in order to have a consistent approach and to be able to make some direct comparisons between numerical results, we are using a unity-based normalization of the waveforms:

$$norm\_s_{k}=\frac{s_{k}-nom\_s_{\text{min}}}{nom\_s_{\text{max}}-nom\_s_{\text{min}}}$$ (14)

where *norm*_*s*_*k*_ is the *k*^*th*^ sample of the normalized waveform, *s*_*k*_ is *k*^*th*^ sample of the original waveform, while *nom*_*s*_min_ and *nom*_*s*_max_ are the minimum and maximum values of the samples of the nominal waveform. As a consequence, all the samples of the normalized nominal waveform lays in the range [0; 1].

### Results for the 1^st^ family

**Fig 5:**
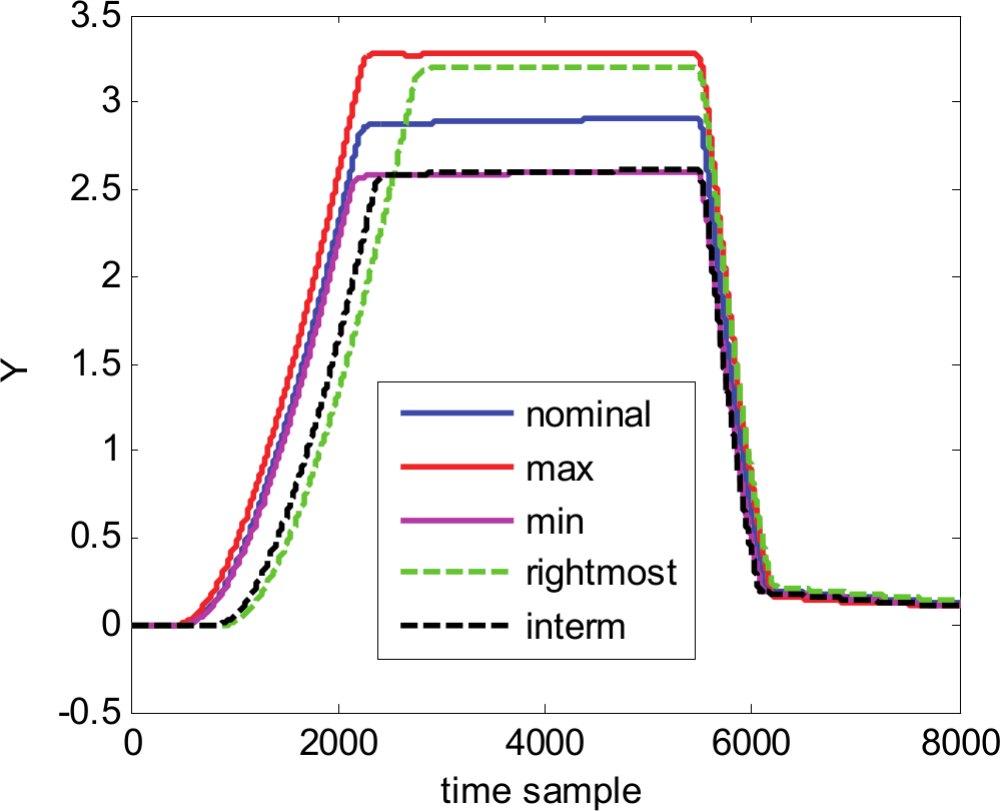
The 1^st^ waveform family, illustrated by the nominal waveform and 4 representative curves.

For the beginning, we start with the 1^st^ waveform family. The original (not-normalized) waveforms in the family are represented in Fig 5 by the nominal waveform and other 4 representative curves: “max”–the waveform with maximum values in the intermediate region; “min”–the waveform with minimum values in the intermediate region; “most right”–the waveform with the most right values in the positive slope region, and “interm”–an intermediate waveform.

#### GA optimization

The first stage in developing the metamodel is to find the optimum wavelet transform and the indices of selected coefficients using the above presented GA optimization.

As previously mentioned, each individual is described by three genes:

- *wavelet_name*, coded by an integer whose value lays in the range [1; 24], each integer indicating a specific wavelet (appropriate for discrete decomposition) in the wavelet families Daubechies, Symlets, and Coiflets, for different order (for example *db*1, *db*3, *sym*2, *sym8*, *coif*1, *coif*3, etc.);
- *decomposition_level*, coded by an integer whose value lays in the range [1; 10], each integer indicating the decomposition level of the wavelet transform;
- *selected_coefficients_ratio*, coded by a positive real number whose value lays in the range [0; 25], indicating the percentage of selected decomposition coefficients.

The objective function (see relation (11)) uses equal weights for the individual objectives, *w*_1_ = *w*_2_ = 1.

Considering the stochastic nature of genetic algorithms, we ran the optimization multiple times, obtaining slightly different final solutions. In Table 1, the optimization results are presented for six solutions denoted *Sol*1, …, *Sol*6. The *mse* values in Table 1 refer to the normalized waveforms (see relation (14)).

**Table 1:** Six solutions for optimum wavelet transform and coefficients selection using GA optimization for the 1^st^ family.

Solution	Wavelet name	Decomposition level	Number of selected coefficients	Selected coefficients ratio	*mse*	
					Nominal waveform	Entire family
*Sol*1	db13	8	142	1.77%	4.7825x10^-9^	2.2972x10^-5^
*Sol*2	db7	8	154	1.93%	8.0138x10^-10^	7.2407x10^-6^
*Sol*3	db13	7	105	1.31%	8.3319x10^-9^	4.3397x10^-5^
*Sol*4	sym4	8	102	1.28%	4.4554x10^-9^	2.2763x10^-5^
*Sol*5	db5	8	111	1.39%	2.1785x10^-9^	1.9336x10^-5^
*Sol*6	db5	6	370	4.63%	3.9386x10^-12^	1.8145x10^-7^

All presented solutions are quite similar from the point of view of accuracy and complexity, providing very good trade-offs between them.

The first 5 solutions (*Sol*1, …, *Sol*5) present an increased accuracy, evaluated by means of *mse* metrics for nominal waveform and also for the entire waveform family (the sum of the individual *mse* for all waveforms in the family). For the nominal waveform, the *mse* is in the range 8.0138x10^-10^ (*Sol*2) to 8.3319x10^-9^ (*Sol*3). Across the entire family, the resulting *mse* (summation for all 200 waveforms) is with 4 orders of magnitude higher than the one for the nominal waveform, lying between 7.2407x10^-6^ (*Sol*2) and 4.3397x10^-5^ (*Sol*3). One can observe that there is a full correlation between the *mse* for the nominal waveform and *mse* for the entire family; the same order of the solution results, regardless of which *mse* (for the nominal waveform or for the entire family) is taken into account, as sorting criterion. This means that the waveform family is a consistent one and the nominal waveform is indeed representative for the family.

From the complexity point of view, the ratio of the selected coefficients is very small, between 1.28% (102 selected coefficients) for *Sol*4 and 1.93% (154 selected coefficients) for *Sol*2. This translates into a substantial data dimensionality reduction, as the ratio between the number of time samples of the waveform and the number of selected coefficients, is 78 for *Sol*4 and 52 for solution *Sol2*.

Solution *Sol*6 in Table 1 provides higher accuracy, because of its better (smaller) values for *mse*, with two orders of magnitude smaller than for the other solutions: 3.9386x10^-12^ for the nominal waveform and 1.8145x10^-7^ across the entire family. The price paid is an increased complexity: 370 selected coefficients (4.63%), that is roughly 3 times greater than in the case of other solutions.

For all presented solutions, the optimum found decomposition level is quite the same (6, 7 or 8). It is worth to remind that the most important (in respect with their magnitude) decomposition coefficients are selected. The coefficients responsible for the low-frequency content of the waveform (namely the approximation coefficients) are entirely selected. Also for the details coefficients, the ratio of the selected coefficients on each decomposition level decreases with the details order. As an illustration, in Table 2, the number of selected coefficients on each level is presented for solution *Sol*5, which involves a db5 wavelet with 8 decomposition levels. For the approximation (*A*_8_), all 40 decomposition coefficients are selected (100%). For the details, the ratio of selected coefficients has a maximum of 57.50% (23 selected out of 40) for *D*_8_, and it decreases with the details order, reaching a minimum of zero for *D*_4_, *D*_3_, *D*_2_, and *D*_1_. In total, 1.38% of the decomposition coefficients were selected, meaning 111 out of a total of 8067 decomposition coefficients.

**Table 2:** Number of selected coefficients for solution *Sol*5, 1^st^ waveform family.

Coefficients	Decomposition level									
	8		7	6	5	4	3	2	1	Total
	*A*_8_	*D*_8_	*D*_7_	*D*_6_	*D*_5_	*D*_4_	*D*_3_	*D*_2_	*D*_1_	
**Resulted from decomposition**	40	40	71	133	258	508	1007	2006	4004	8067
**Selected**	40	23	23	20	5	0	0	0	0	111
**Ratio of selected [%]**	100	57.50	32.39	15.04	1.94	0	0	0	0	1.38

**Fig 6:**
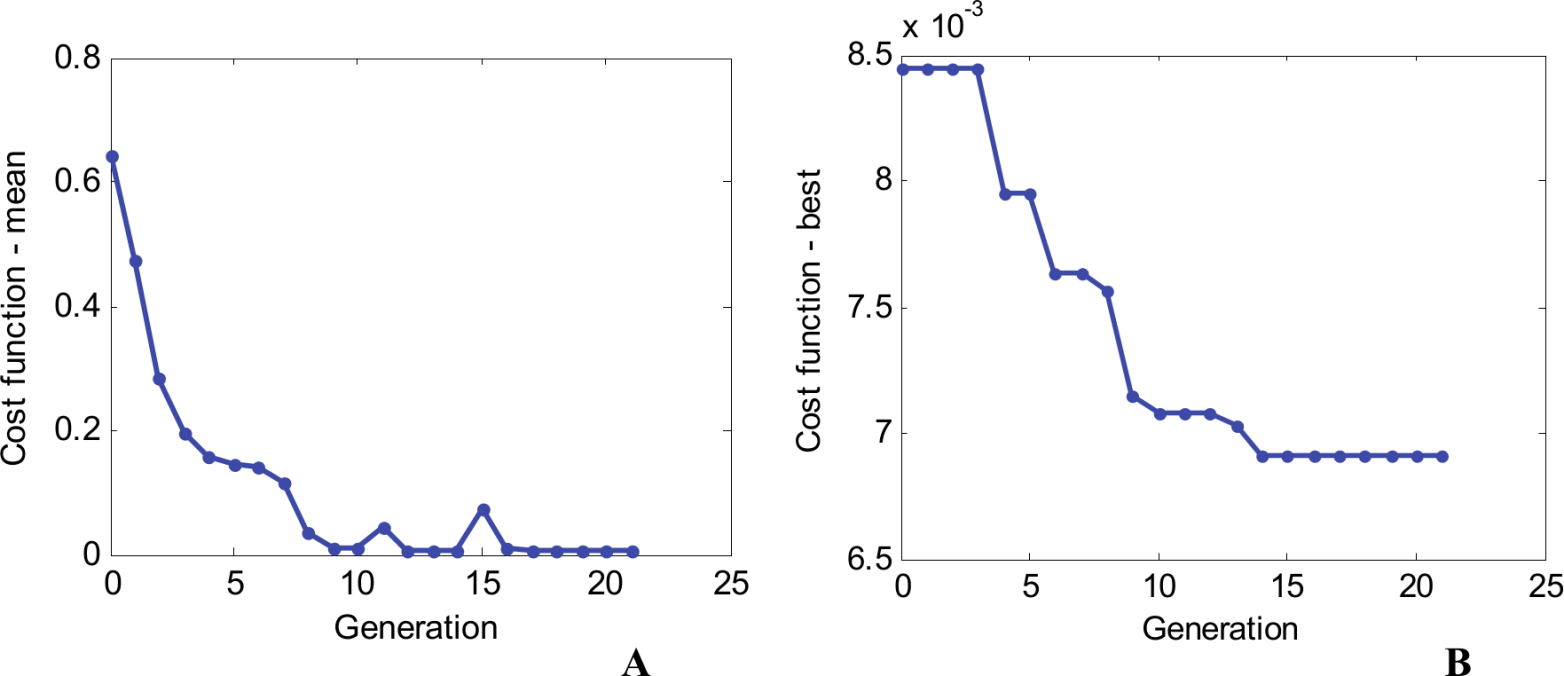
Cost function evolution during the optimization for *Sol*5, 1^st^ waveform family. (A) Mean value across the entire population. (B) Best individual.

Some relevant information about the dynamics of the GA optimization is presented for solution *Sol*5. The evolution of the cost functions (mean and best values) is presented in Fig 6. One can notice a global improvement of the whole population (30 individuals split into two subpopulations) by a global decrease of the mean value of the cost function, from 0.642 for the initial population, down to 7.0x10^-03^, in the last generation (21). Also, the cost function for the best individual in each generation improves constantly, from an initial value of 8.445x10^-3^, down to the final value of 6.908x10^-3^ in generation 14. On the last 7 generations (from 14 to 21), no further improvement of the cost function (best individual) appears and the optimization stops (stall generations was set to 7).

To evaluate the computational effort required for developing the metamodel, we mention here that the experiments were conducted on a general purpose computer (i5-4460 CPU @ 3.2GHz, 8GbRAM, 64-bit operating system). The time necessary to run the GA optimization has a medium value (across the above presented solution) of 11 min, with a maximum of 17 min for *Sol*1 (27 generations). For the solution we use further (*Sol5*), the optimization time was 10 min.

#### ANN generation and training

Once we know the optimum wavelet transform (mother wavelet and decomposition level) for the waveform family under consideration, the next step for metamodel development is the generation of the data set for the neural network architecture selection and training. In this section, we illustrate this part using the above generated solution *Sol*5.

The output data (targets) is organized in the *SC* matrix (see relation (12)) with *N* = 200 columns and *Q* = 111 rows, by applying the wavelet decomposition and coefficients selection for all 200 waveforms in the waveform family. The input data is organized in the *P* matrix (see relation (13)) with *N* = 200 columns and *M* = 10 rows.

**Fig 7:**
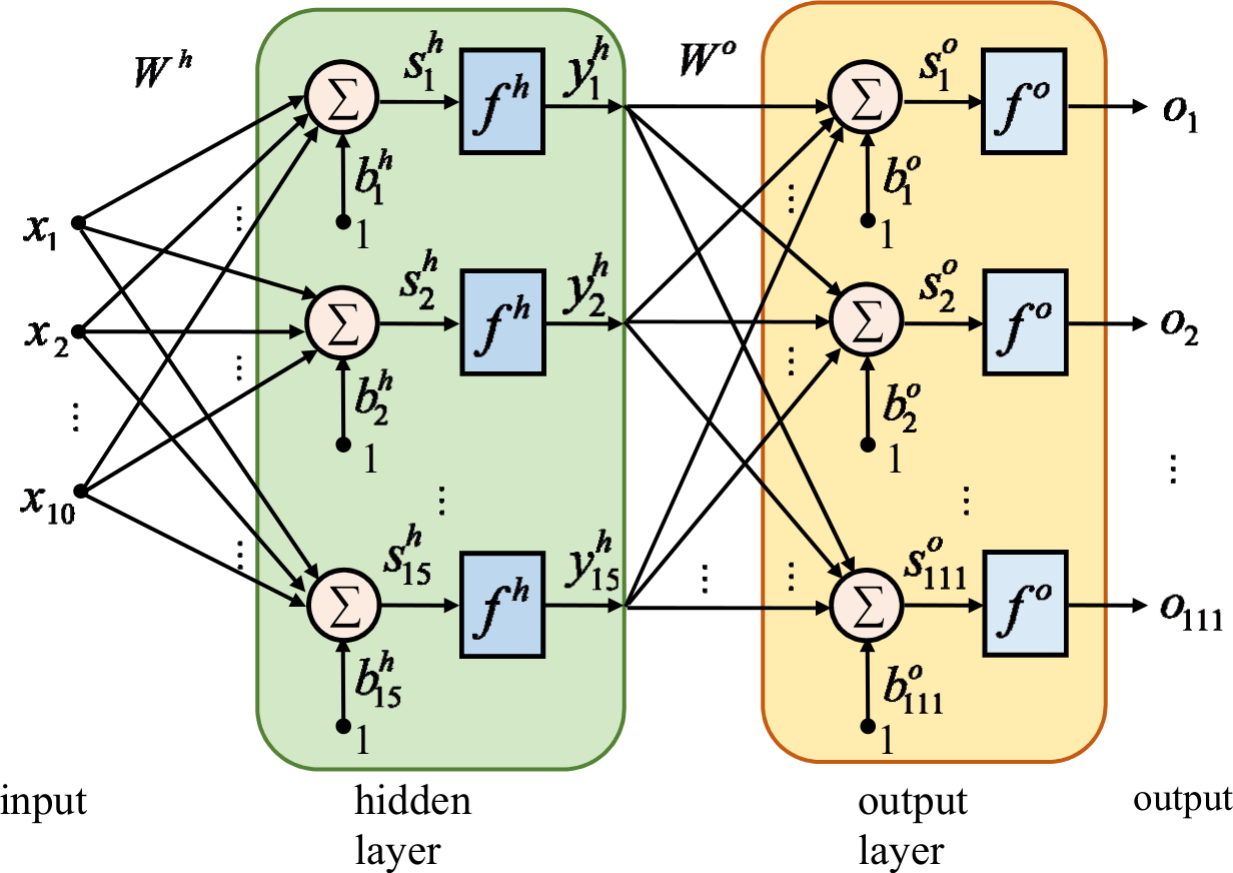
Architecture of the ANN of the metamodel for the 1^st^ waveform family.

We use a neural network whose architecture is presented in Fig 7. The network presents:

- 10 inputs (*x*_1_, *x*_2_, …, *x*_10_) - the number of input parameters;
- 111 outputs (*o*_1_, *o*_2_, …, *o*_111_) - one output for each (selected) coefficient, meaning that the output layer presents 111 neurons;
- one hidden layer, composed by a number of 15 neurons; the number of neurons was chosen after a series of trial runs.

The other notations used in Fig 7 are:

- *W*^*h*^ - the matrix of weights for the hidden layer;
- ∑ - the summing block inside of each artificial neuron;
- $b_{1}^{h},\;b_{2}^{h},\;\ldots ,\;b_{15}^{h}$ - the biases for the neurons in the hidden layer;
- $s_{1}^{h},\;s_{2}^{h},\;\ldots ,\;s_{15}^{h}$ - the outputs of the summing blocks for the neurons in the hidden layer;
- *f*^*h*^ - the pure linear activation function for the neurons in the hidden layer;
- $y_{1}^{h},\;y_{2}^{h},\;\ldots ,\;y_{15}^{h}$ - the outputs of the neurons in the hidden layer;
- *W*^*o*^ - the matrix of weights for the output layer;
- $b_{1}^{o},\;b_{2}^{o},\;\ldots ,\;b_{111}^{o}$ - the biases for the neurons in the output layer;
- $s_{1}^{o},\;s_{2}^{o},\;\ldots ,\;s_{111}^{o}$ - the outputs of the summing blocks for the neurons in the output layer;
- *f*^*o*^ - the sigmoidal activation function for the neurons in the output layer.

For the training procedure, the full data set (200 data pairs) is split into three data subsets: training subset (90% of the data set), validation and testing subsets 10% (5% for validation and 5% for testing).

Training multiple times will generate different results due to different initial conditions (initiation of weights and biases with randomly generated values) and data sampling [35]. In our implementation, the procedure involves multiple training trials, and the neural network that presents the highest regression value *R* across the entire data set is chosen as a final solution. The regression measures the correlation between outputs and targets. An *R* value of 1 is the best, meaning a perfect fit between outputs and targets, while 0 means no correlation (random relationship). Table 3 presents some results for 8 training trials.

**Table 3:** Results for 8 ANN training trials, *Sol*5, 1^st^ waveform family.

Performance metrics	ANN training trial							
	01	02	03	04	05	06	07	08
**Regression**	.999949	.999945	.999943	.999949	.999954	.999959	.999944	.999949
**Training *mse***	2.7x10^-3^	2.9x10^-3^	3.2x10^-3^	2.7x10^-3^	2.5x10^-3^	2.3x10^-3^	3.1x10^-3^	2.8x10^-3^
**Validation *mse***	3.0x10^-3^	5.6x10^-3^	3.2x10^-3^	3.2x10^-3^	2.0x10^-3^	1.9x10^-3^	3.3x10^-3^	3.6x10^-3^
**Testing *mse***	6.7x10^-3^	4.2x10^-3^	4.4x10^-3^	4.9x10^-3^	4.7x10^-3^	2.3x10^-3^	4.3x10^-3^	3.7x10^-3^
**Epochs**	404	375	380	659	729	809	402	419

Even if the starting point for training (initial values of weights and biases of ANN) is different and the data assignment into the three data sub-sets is also different for every trial, the results presented in Table 3 confirm that the training process is a robust one and it is always convergent, producing neural networks with almost the same final performances. The regression value *R* indicate a very high accuracy of the ANN, laying between 0.999959 (trial 06) and 0.999943 (trial 03).

The *mse* between the original coefficients (targets) and the coefficients computed by the ANN (outputs) is quite similar for all trials in the training data set (between 2.3x10^-3^ for trial 06 and 3.2x10^-03^ for trial 03) but also in the validation and testing data sets considered together (1.9x10^-3^ in trial 06 for validation and 6.7x10^-3^ in trial 01 for testing). Conclusively, irrespective of initial condition and data sampling, the resulted ANN presents a high level of accuracy.

**Fig 8:**
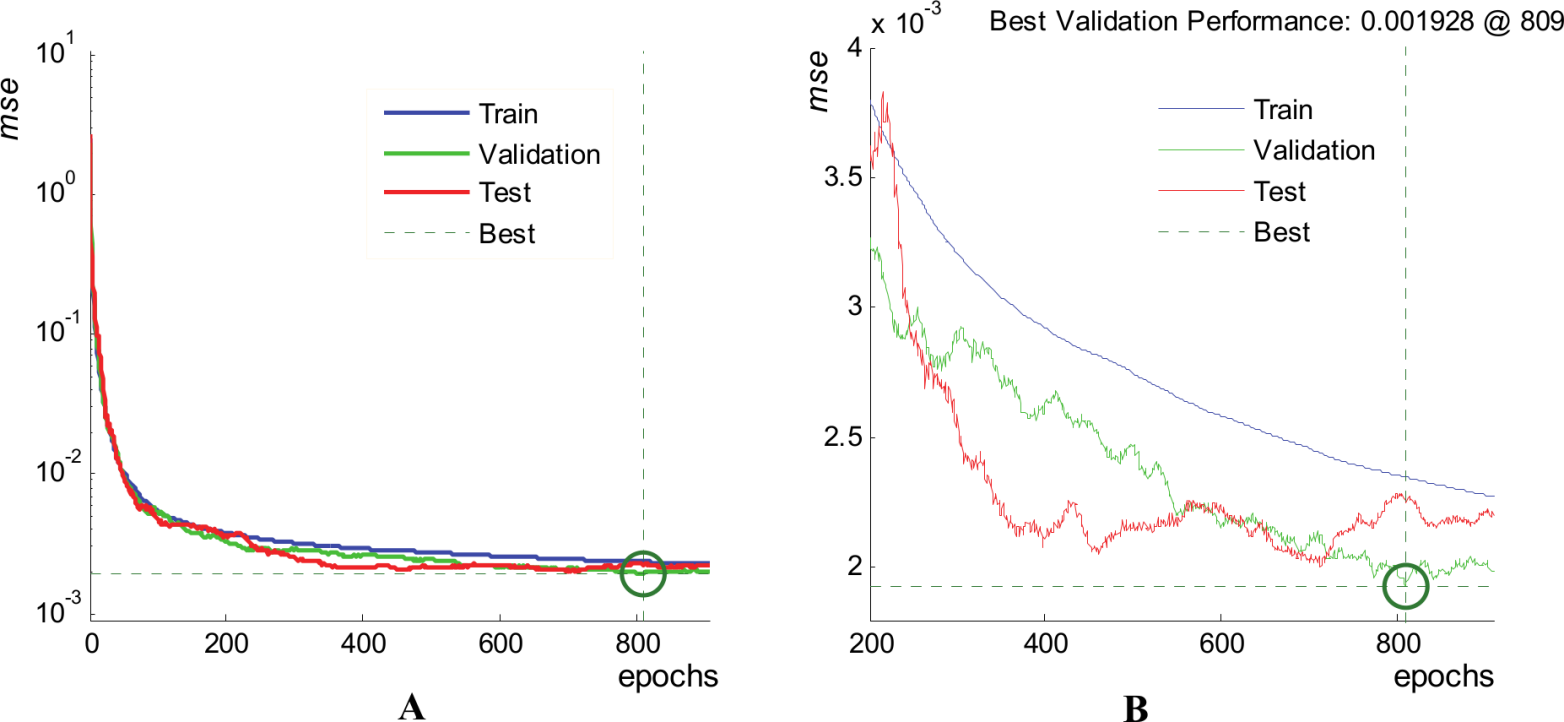
Performance validation graph for ANN training trial 06, *Sol*5, 1^st^ waveform family. (A) All training epochs (log scale for *mse*). (B) Details for fine tuning epochs: 200 to 909 (linear scale for *mse*).

Hereinafter we illustrate the ANN training process and detailed performances for ANN training trial 06, *Sol5*. The performance validation graph is presented in Fig 8. It depicts the evolution of the mean squared error within the three subsets. Fig 8A uses a logarithmic scale on the *mse* axis to accommodate the large variation of *mse* (over three magnitude order) during the full optimization process. In the first 200 training epochs, one can see a steep improvement (lowering-in) of the *mse*s in all data subsets. Then, the training enters the phase of “fine tuning”, continuously improving the performance, as it can be observed on Fig 8B that uses a linear scale to plot details on *mse* evolution. The training ends after 909 epochs, but the best trained ANN is considered to be the one at iteration 809, where it presents the best validation performance (1.928x10^-3^). It is observed that after epoch 809 the overfitting phenomenon appears, the *mse* in the validation subset (green curve) having a tendency to slightly increase on the next 100 consecutive epochs.

**Fig 9:**
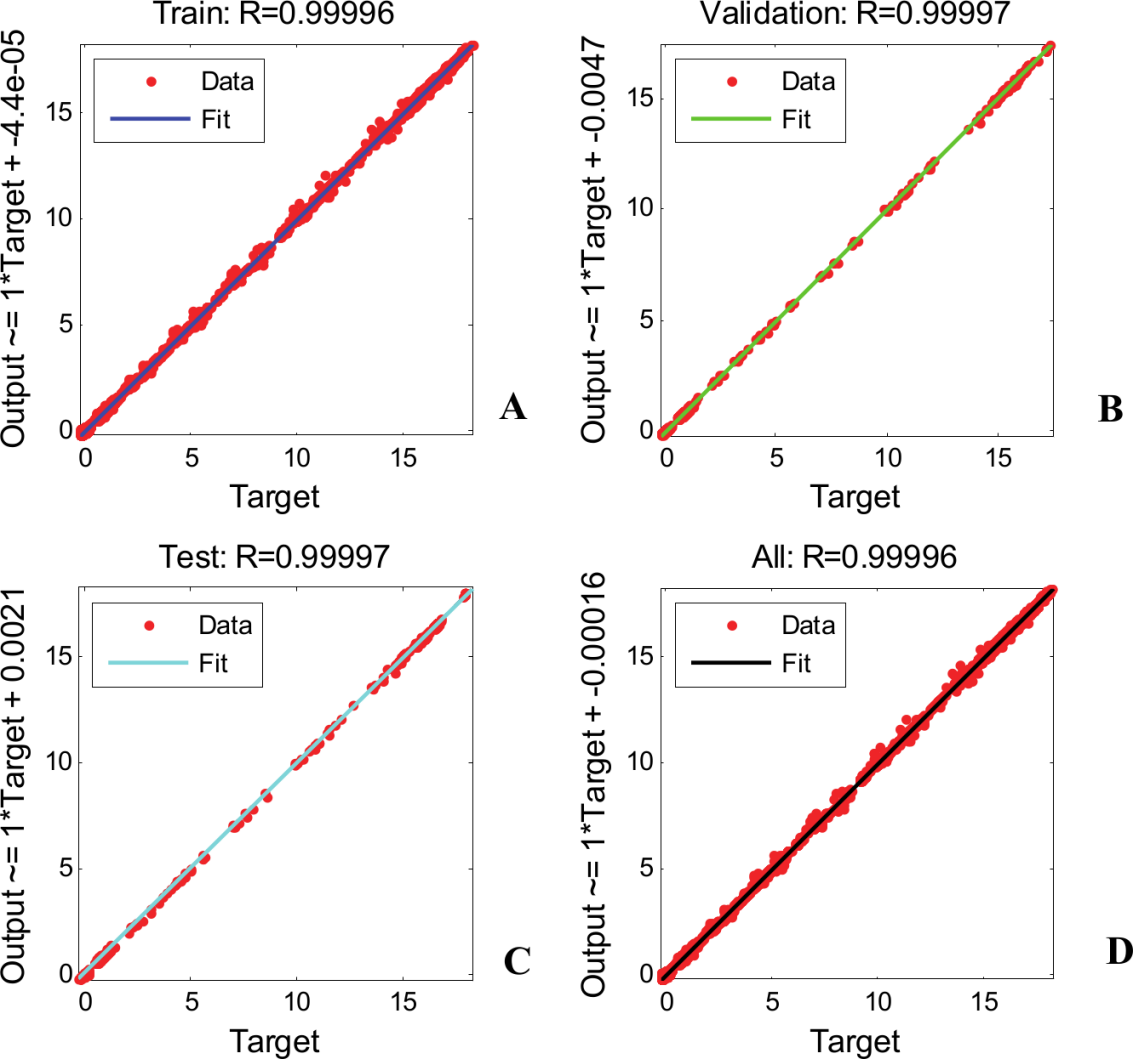
Linear regression in all data subsets for ANN training trial 06, *Sol*5, 1^st^ waveform family. (A) Training subset. (B) Validation subset. (C) Testing subset. (D) All data.

The trained neural network presents excellent fitting performances in all data subsets. This can be seen by inspecting the results for linear regression of outputs of the neural network (predicted values) relative to the targets (original values) presented in Fig 9. A measure of the “goodness of fit” is the regression value, *R*. The linear regression equation is:

Output=a⋅Target+b (15)

where *a* is the slope of regression fit and *b* is the offset of regression fit.

An ideal fit (network outputs match the targets exactly) would presents *R* = 1, *a* = 1 and *b* = 0. The slope of the regression equation is *a* = 1 in all data subsets and the offset is very close to zero, with a maximum value of 4.7x10^-3^ in the validation subset and a value of 1.6x10^-4^ across all data. The regression value, *R*, is a very good one as well: 0.99996 in the training subset and across all data and 0.99997 in the validation and testing subsets.

Fig 9 also provides a qualitative appreciation of the ANN accuracy. The ideal result should place all data points on the first bisector. In our case, almost all data points are correctly placed on the first bisector, with only few points slightly shifted from their ideal position.

The linear regression offers information on two extremes. On the one hand it provides a global appreciation of the accuracy (through the regression value *R* and through the slope *a* and offset *b* of the regression equation), but on the other hand it compares the position of each generated data point with its target counterpart. The global appreciation can “hide” some errors, while the detailed plot of each point can lead to a very difficult to analyze image. For example, in the testing subset (Fig 9C) 1110 data points are represented (111 outputs of the ANN, with 10 value each).

**Fig 10:**
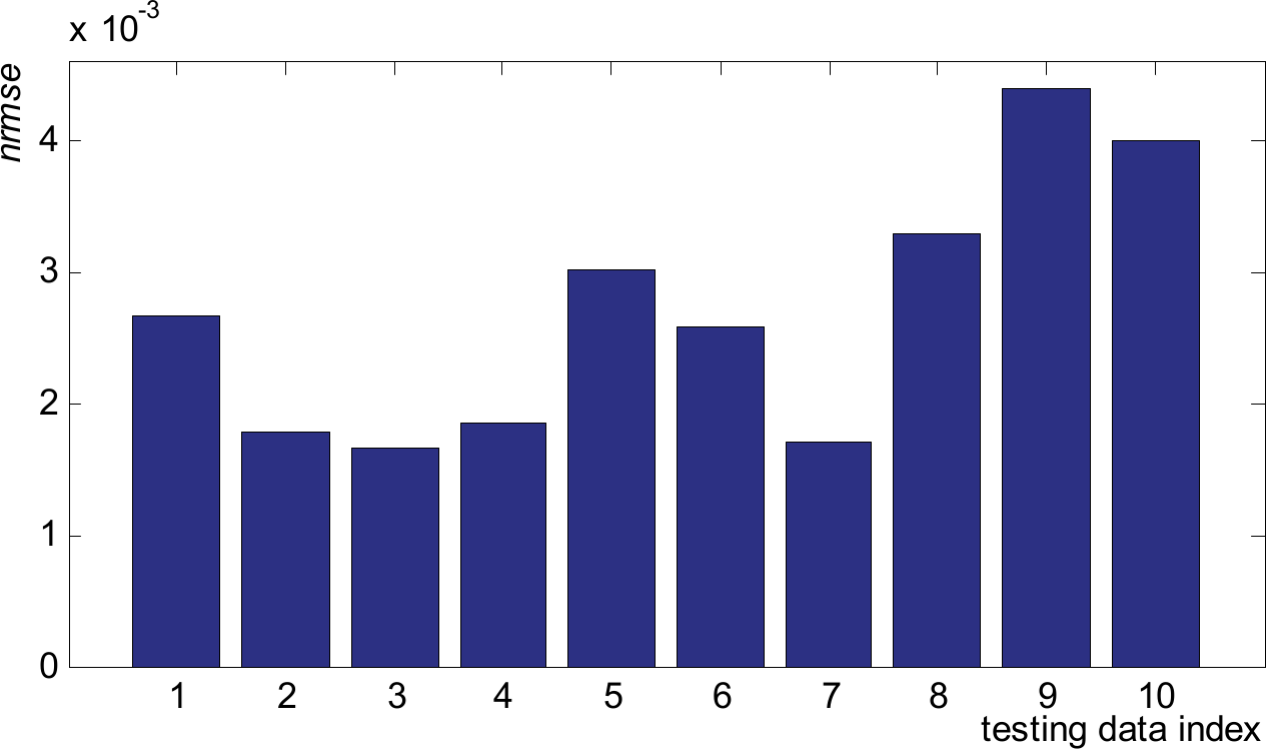
Values of *nrmse* for the 10 sample data points (waveform) in the testing subset.

To fully describe the results, beside the linear regression, we will use some intermediate error representations. Fig 10 presents the normalized root mean squared error (*nrmse*) computed for the 10 sample data points (waveforms) in the testing subset. The relation to determine *nrmse* is:

$$nrmse=\frac{\frac{1}{Q}\sum_{i=1}^{Q}{\left(o_{i}-t_{i}\right)}^{2}}{t_{\text{max}}-t_{\text{min}}}$$ (16)

where *Q* is the number of ANN outputs, *o*_*i*_ is the *i*^*th*^ generated output, *t*_*i*_ is the *i*^*th*^ target, *t*_max_ is the maximum value of target values, and *t*_min_ is the minimum value of target values.

**Fig 11:**
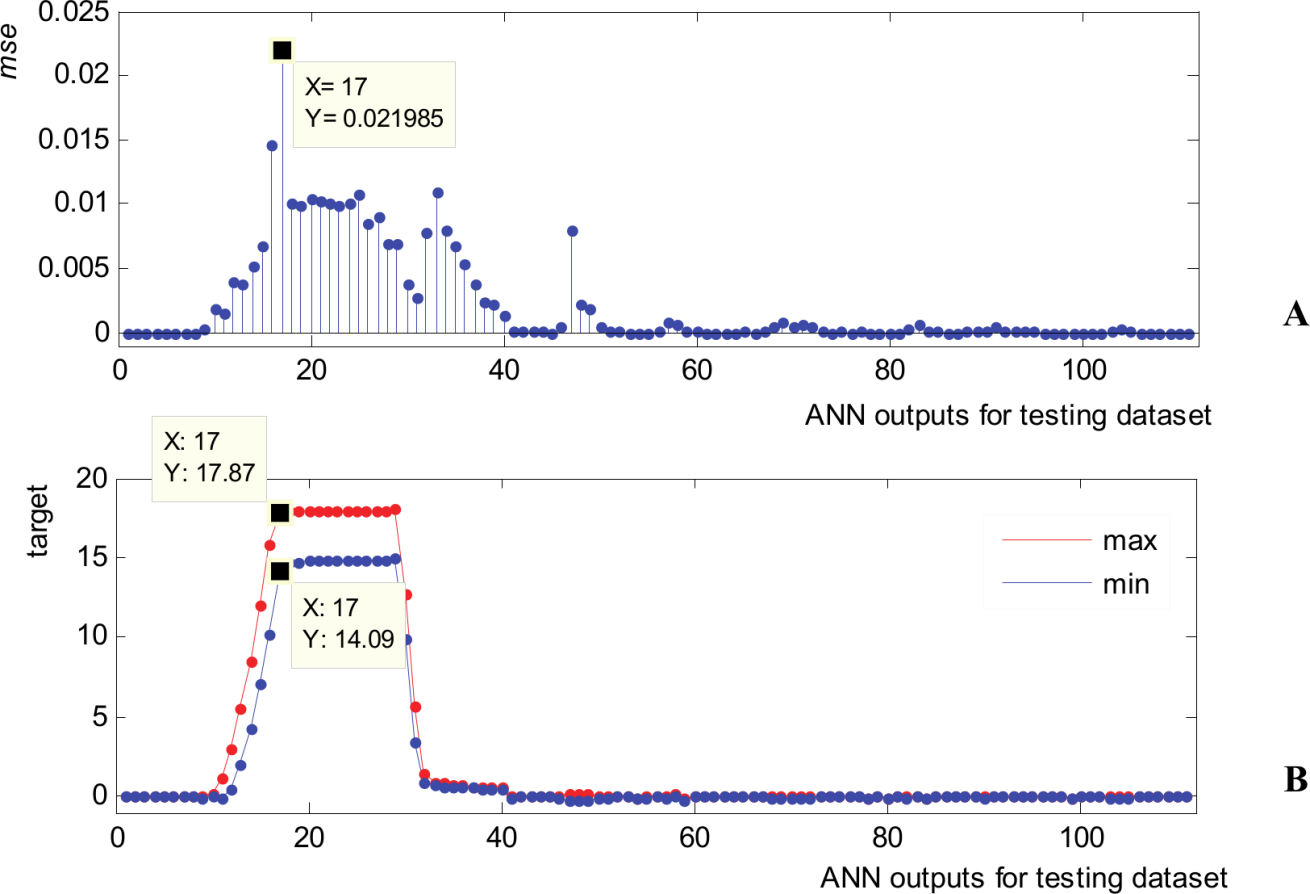
Prediction accuracy of ANN for testing data subset. (A) *Mse* error for ANN outputs (111 outputs). (B) Dynamic interval of targets: max values (red) and min values (blue).

For all 10 testing waveforms the *nrmse* is very small, laying in the range of 1.67x10^-3^ for waveform 3 and 4.39x10^-3^ for waveform 9. It results that the ANN presents high accuracy and very good generalization (prediction) capability for all waveforms in the testing subsets, as these waveforms was not at all involved in the training process. To characterize the ANN from the point of view of its individual outputs, Fig 11 presents the mean squared error encountered for the testing subset for each of the 111 outputs. The largest *mse* values (Fig 11A) happens for the target presenting the largest values and widest dynamic range (Fig 11B). These correlation is normal and it is mainly justified by the fact that *mse* is scale dependent. The maximum *mse* (0.021985) appears on output 17 where the target presents maximum value and maximum dynamic range (from 17.87 down to 14.09).

From the point of view of the training time, the total time for training (8 trials) and selecting the final solution for the ANN is less than 1min, small enough to arise no issues regarding the metamodel development time. Referring to the total time necessary for the metamodel development, once the waveform family is available, the most time consuming tasks are the GA optimization and ANN training. Considering the previously mentioned consumed time, a maximum of 17 min for GA optimization and 1 min for ANN training, results in a total time of maximum 18 min on a general purpose computer. It is obvious that the computational effort required for constructing the metamodel is absolutely affordable.

The final step in our metamodel implementation refers to the metamodel integration, meaning (according to Fig 1) to fusion the coefficients generated by the ANN with the remaining necessary coefficients (taken form the nominal waveform decomposition), in full accordance with the indices of the selected coefficients. To generate the output waveform by its time samples, the wavelet reconstruction is applied on the full coefficients vector, using the same wavelet transform (wavelet and bookkeeping vector) stored in the knowledge database.

**Fig 12:**
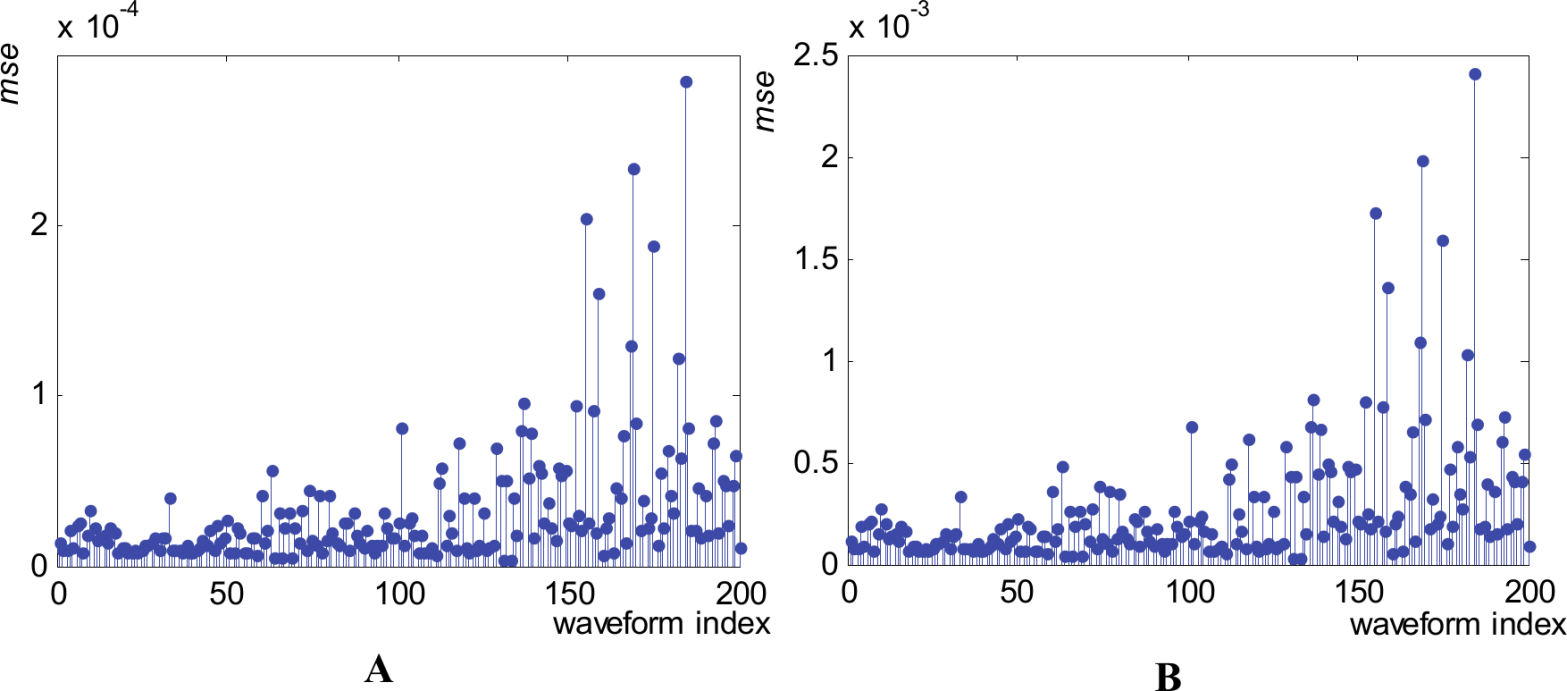
Mean squared errors (*mse*) obtained using the metamodel for the generation of all 200 waveforms in the 1^st^ waveform family. (A) Normalized waveforms. (B) Original waveforms (without normalization).

In order to appreciate the accuracy of the resulted metamodel, we apply it to all our 200 available parameter combinations. Fig 12 presents the value of the “goodness of fit”, meaning *mse* for all generated waveforms, for both normalized and original waveform versions.

The mean value of *mse* across the entire family is 3.1837x10^-5^ in the normalized version and 2.693x10^-4^ for the original version. All individual and mean values of *mse* indicate a high accuracy of our metamodel. The distribution of the *mse* values across the entire waveform family, meaning few cases with relatively high values, few cases with medium values, a lot of cases with low values, and no cases with zero values, indicates that our metamodel present very good generalization capabilities.

The top three low accuracy (greater *mse*) generated waveform are presented in Table 4. The greatest *mse* is encountered for waveform 184 (2.8418x10^-4^ for normalized version, respectively 2.4038x10^-3^ in the original version). Even for this extreme case, the accuracy is quite high: the correlations between the original waveform and the predicted one are very good, as the value of the regression factor is very close to 1, *R* = 0.9993.

**Table 4:** Top three low accuracy generated waveforms, 1^st^ waveform family.

Waveform index	*mse* (normalized)	*mse* (original)
184	2.8418x10^-4^	2.4038x10^-3^
169	2.3361x10^-4^	1.9759x10^-3^
155	2.0471x10^-4^	1.7315x10^-3^

On the other hand, the minimum value of the *mse* is encountered for waveform 131, being 3.0273x10^-6^ for the normalized version and 2.5607x10^-5^ for the original version.

**Fig 13:**
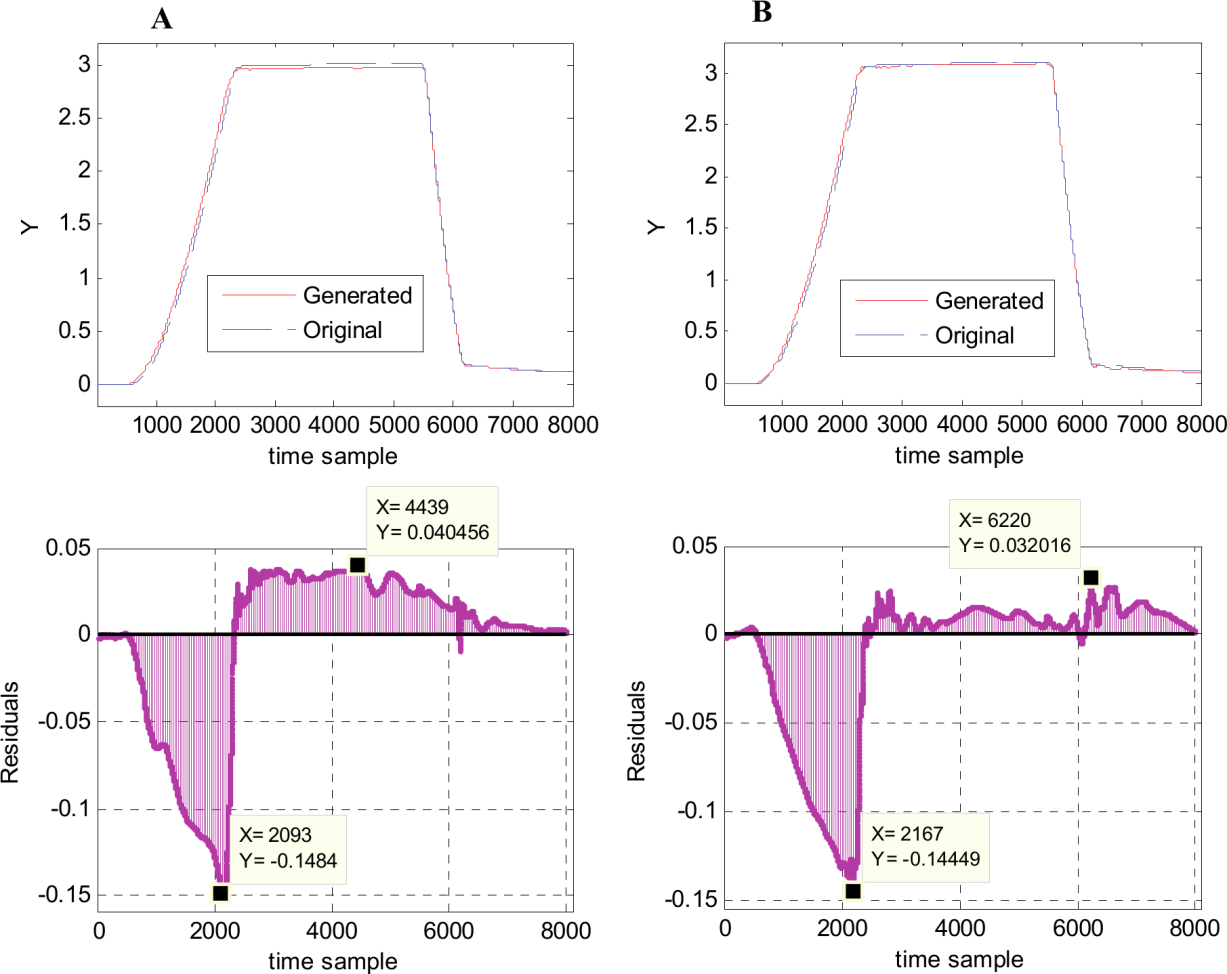
Comparison between the generated and original waveforms in two cases. (A) Highest *mse*–waveform 184 (generated vs. original waveform–top; residuals—bottom). (B) Second highest *mse*–waveform 169 (generated vs. original waveform–top; residuals—bottom).

Another perspective for the metamodel accuracy is offered by the plots in Fig 13, which depict a direct comparison between the generated and original waveforms, in two cases: waveform 184, that presents the highest *mse* value and waveform 169, which presents the second highest *mse* value. In both cases, the generated waveforms are almost identical with the original ones, with only few differences. For waveform 184, the differences appears in the region with positive slope, between samples 600–2300 and in the region with almost constant high values (samples 2300–5000), as one can see in Fig 13A top. The residuals are presented in Fig 13A bottom. The residuals are mainly negative in the first region (positive slope) with a maximum magnitude of 0.1484 for sample 2093, and positive in the rest with a maximum magnitude of 0.040456 at sample 4439. For waveform 169 the differences between generated and original waveforms are mainly presented in the region of positive slope (samples 600–2350), see Fig 13B top. The associated residuals (Fig 13B bottom) are negative in that region, with a maximum magnitude of 0.14449 at time sample 2167. In the remaining part of the waveform, the residuals are positive, with a maximum magnitude of 0.032016 at time sample 6220.

By computing the sample-by-sample error (residuals), in fact one computes the variation on the horizontal axis, thus obtaining a larger error that it really is, considering the entire region in discussion. It is obvious that comparing two waveforms using the sample-by-sample difference on the horizontal axis is not the best approach, at least not on the regions presenting steep slopes. Hence, another measure to compare two waveforms should be developed, but it is outside of the scope of the present work.

It is worth to mention here that the *mse* in the worst case (2.4038x10^-3^ for waveform 184) is with two magnitude orders greater than the *mse* for the best case (2.5607x10^-5^ for waveform 131). This is due to the fact that in the available data set (200 waveforms) there are a reduced number of examples similar with waveform 184. The solution for better metamodel accuracy as a whole is to obtain and use a larger data set for developing (training) the metamodel.

**Fig 14:**
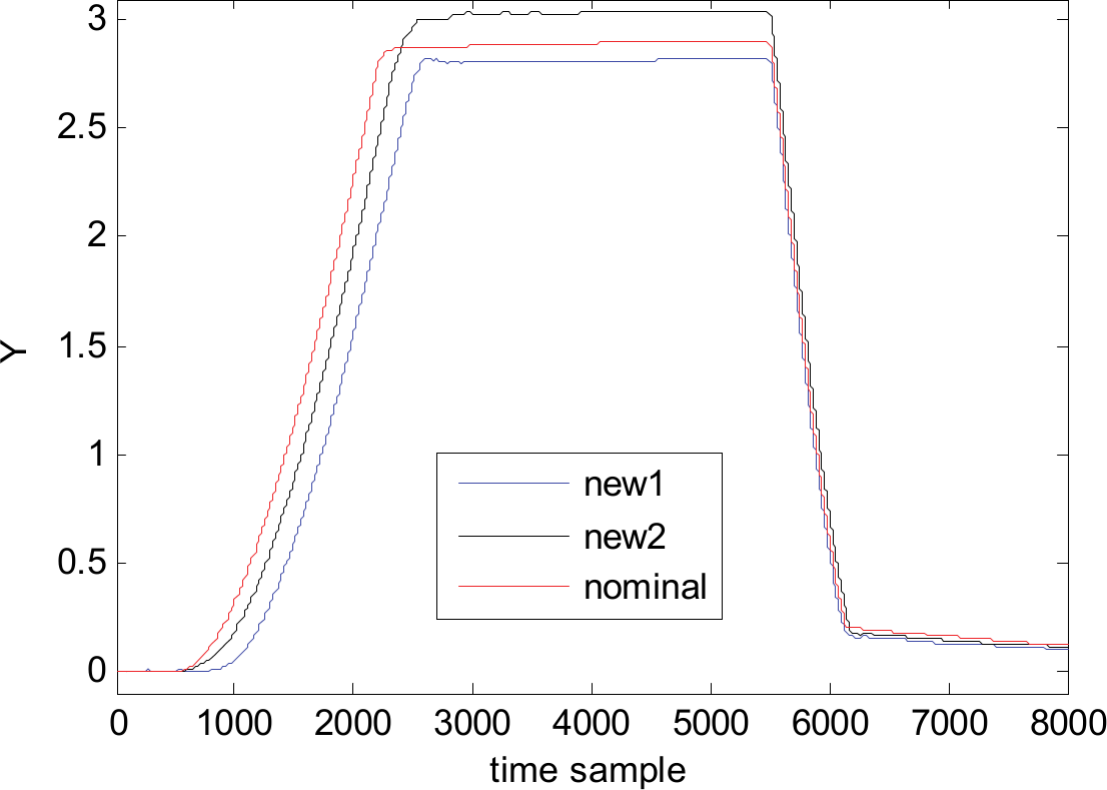
Two new generated waveforms and the nominal waveform (1^st^ waveform family).

To better understand and appreciate the quality of the metamodel we are using it to generate new waveforms for completely different parameter combinations. The new values of the parameters are randomly generated and checked to be different from the 200 combinations that were already used for building the metamodel. As an illustration, Fig 14 presents two such waveforms, denoted new1 and new2. For the sake of comparison, the nominal waveform is also plotted on the same figure. According with the shape and values of new waveforms, compared with the nominal one and with the other waveforms of the family (see Fig 5) it is clear that they belong to the 1^st^ waveform family, so the metamodel can be credited as a very reliable one.

### Results for the 2^nd^ and 3^rd^ families

**Fig 15:**
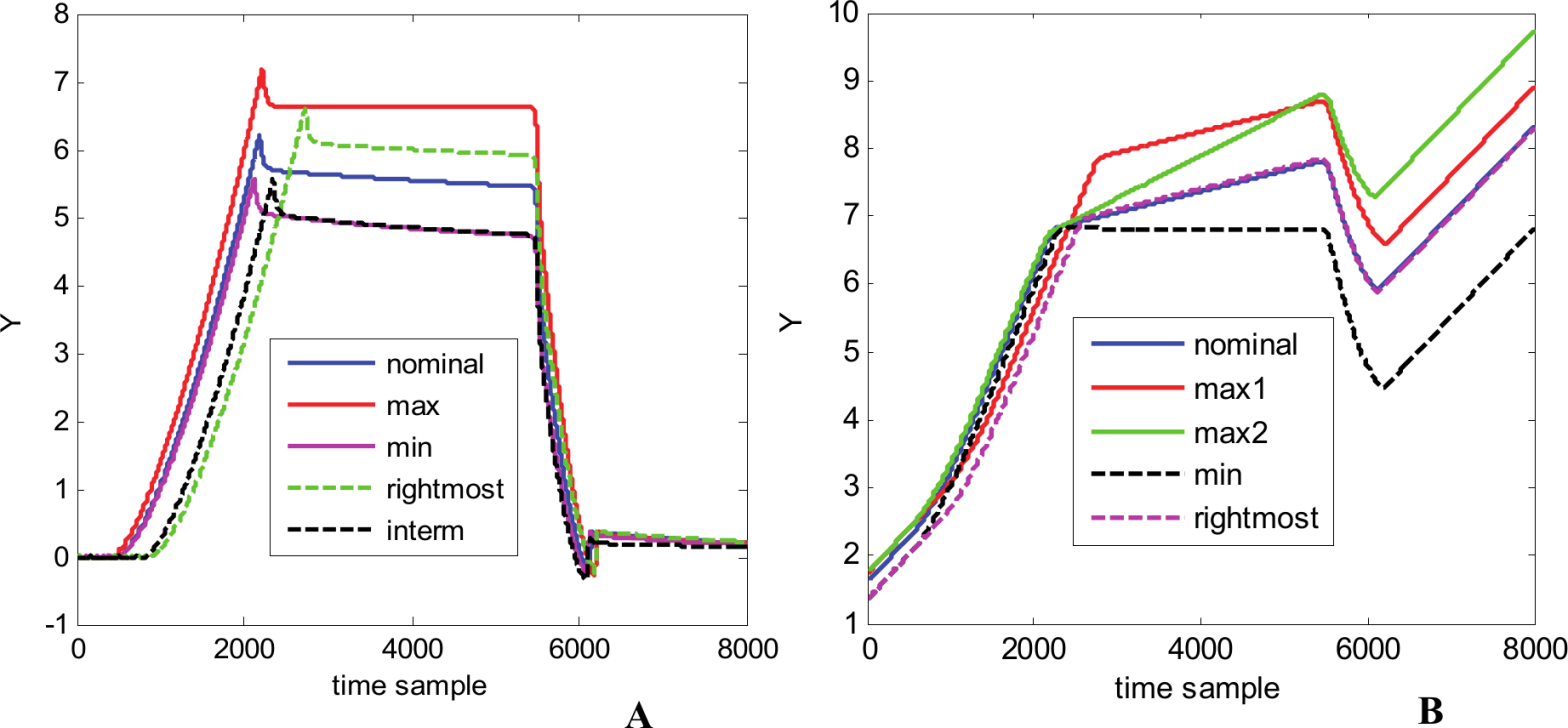
Waveform families illustrated by the nominal waveform and 4 more representative curves. (A) 2^nd^ waveform family. (B) 3^rd^ waveform family.

Hereafter, it follows the presentation and discussion of experimental results for the 2^nd^ and 3^rd^ waveform families. The 2^nd^ family is presented in Fig 15A through the nominal waveform and other 4 representative curves: “max”–the waveform with maximum values in the intermediate region; “min”–the waveform with minimum values in the intermediate region; “rightmost”–the waveform with the rightmost values in positive slope region, and “interm”–an intermediate waveform. Compared with the 1^st^ family (see Fig 5) the 2^nd^ family appears to be more complex, each waveform presenting an overshot at the end of the rise time and also at the end of the fall time.

Fig 15B presents the 3^rd^ waveform family through the nominal waveform and other 4 representative curves: “max1”–the waveform with maximum values in the intermediate region (samples 2250–5500); “max2”–the waveform with maximum values in the right side region (samples 5500–8000); “min”–the waveform with minimum values in the intermediate and left side regions (samples 2500–8000), and “rightmost”–the waveform with the rightmost values in the positive slope region (samples 0–2500). One can remark that the waveforms in this family present large variations of sample values inside of each individual waveform but also from one waveform to another, especially in its second half.

Using the same settings for the GA optimization as the ones used for the 1^st^ family, we run the optimization six times for each waveform family. Table 5 contains the final optimal solutions for wavelet transform and coefficients selections for both families.

**Table 5:** Optimal solutions for wavelet transform and coefficients selection using GA optimization for the 2^nd^ and 3^rd^ waveform families.

Waveform family	Wavelet name	Decomposition level	Number of selected coefficients	Selected coefficients ratio	*mse*	
					Nominal waveform	Entire family
2^nd^	sym7	8	239	2.83%	5.8499x10^-9^	4.2533x10^-5^
3^rd^	sym3	7	181	2.26%	7.5886x10^-10^	8.1433x10^-6^

The optimal solutions in both families are quite alike: same type of mother wavelet (sym7, respectively sym3); 8, respectively 7 decomposition levels; 239, respectively 181 selected coefficients.

From the complexity point of view, the ratio of the selected coefficients is small, 2.83% (239 selected coefficients) for the 2^nd^ family and 2.26% (181 selected coefficients) for the 3^rd^ family. This translates into an important data dimensionality reduction, the ratio between the total number of waveform samples and the number of selected coefficients being 33.5 for the 2^nd^ family and 44.4 for the 3^rd^ family. The accuracy of approximating the 8000 time samples waveforms by a drastically reduced number of wavelet coefficients is a very increased one for nominal waveforms (5.8499x10^-9^, respectively 7.5886x10^-10^), but also across the entire family (4.2533x10^-5^, respectively 8.1433x10^-6^).

Compared with the results obtained for the first family (see Table 1), the optimum found wavelet transforms are similar from the point of view of decomposition level (7 and 8). It can be noticed that, even if the number of time samples is identical for all families (8000), for the 2^nd^ and 3^rd^ families the number of selected coefficients and the selected coefficient ratios are larger than in the case of the 1^st^ family, to obtain similar *mse* (same magnitude order), for both nominal waveforms and across the entire family. This is a direct consequence of the fact that the complexity of the waveforms in the 2^nd^ and 3^rd^ families is greater than the waveform complexity in the 1^st^ family (compare Figs 5 and 15).

The necessary time to run the GA optimization for the 2^nd^ waveform family has a medium value (across the six optimization trials) of 8 min and 30 s, with a maximum of 11 min for one trial. For the 3^rd^ waveform family the times are similar: medium time of 8 min and a maximum time of 13 min for one trial.

To complete the metamodel development, the neural network that maps the input parameter combination into the selected coefficients value has to be generated and trained. The architecture of the ANNs is similar with the one presented in Fig 7. For each family, 8 training trials took place in the quest of the final ANN. Table 6 presents the final results for each family, both neural network topology and training performances. For the 2^nd^ family there are 10 inputs, 13 neurons in the hidden layer, and 239 neurons in the output layer. The ANN for the 3^rd^ waveform family presents 10 inputs, 16 neurons in the hidden layer, and 181 neurons in the output layer.

**Table 6:** ANN topologies and training performances for 2^nd^ and 3^rd^ waveform families.

Waveform family	ANN topology			Training performances				
	Inputs	Hidden layer neurons	Outputs	Regression	Training *mse*	Validation *mse*	Testing *mse*	Epochs
2^nd^	10	13	239	.999898	2.5x10^-3^	2.7x10^-3^	2.0x10^-3^	331
3^rd^	10	16	181	.999970	1.1x10^-3^	5.5x10^-4^	5.8x10^-4^	896

The results confirm that the training process is a robust one, always convergent, producing neural networks with almost the same final performances. The regression value *R* indicate a very high accuracy of the trained ANNs being 0.999898 (2^nd^ family) and 0.999970 (3^rd^ family). In the case of the 2^nd^ waveform family the *mse* between original coefficients (targets) and the coefficients computed by the ANN (outputs) is quite similar in the training data subset 2.5x10^-3^ and in the validation and testing data subsets (2.7x10^-3^, respectively 2.0x10^-3^). For the 3^rd^ family there is a small difference, the *mse* in the validation and testing subsets being approximately half of the one in the training subset.

The time necessary for training (8 trials) and selecting the final ANN for integration in the metamodel was approximately 30 s for the 2^nd^ family and slightly below 1 min for the 3^rd^ family. Completely, the total time necessary to develop the metamodel (in the worst case) is less than 12 min (maximum 11 min for GA optimization and 30 s for ANN training) for the 2^nd^ family and less than 14 min (maximum 13 min for GA optimization and 1 min for ANN training) for the 3^rd^ family.

**Fig 16:**
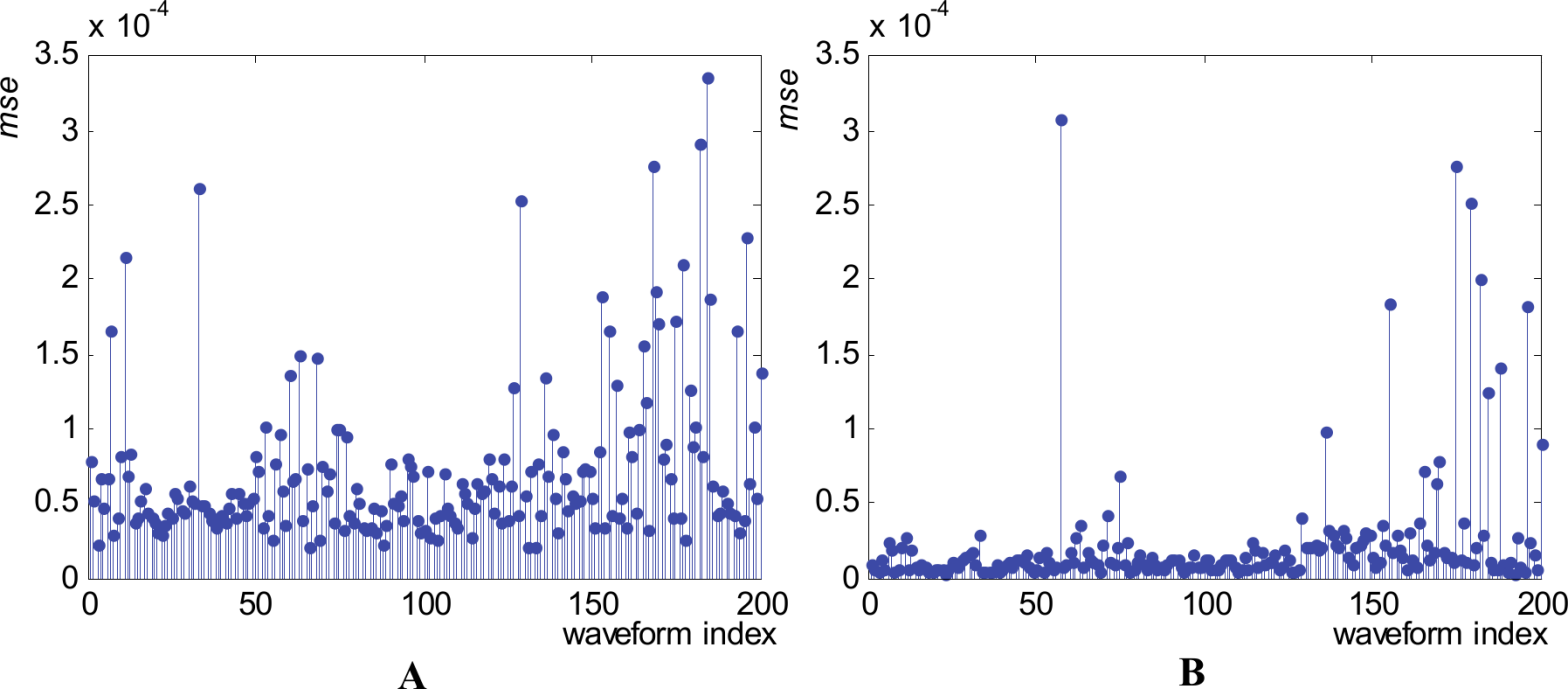
Mean squared errors (*mse*) obtained using the metamodel for the generation of the normalized waveforms. (A) 2^nd^ family. (B) 3^rd^ family.

To globally appreciate the accuracy of our metamodels we apply them for all our 200 available waveforms in each family. Fig 16 presents the value of the “goodness of fit”, meaning *mse* for all waveforms, for normalized waveforms.

The mean value of *mse* is 7.0648x10^-5^ across the 2^nd^ family and 2.2924x10^-5^ across the 3^rd^ family. All individual and mean values of *mse* indicate high generation accuracy of our metamodels. The lowest accuracy (greater *mse*) generated waveforms from each family are the ones presented in Table 7. The greatest *mse* occurs for waveform 184 (3.3616x10^-4^, in the normalized version and 1.4057x10^-2^ in the original version) in the 2^nd^ family and for waveform 57 (3.0750x10^-4^ in the normalized version and 1.3853x10^-2^ in the original waveform) in the 3^rd^ family.

**Table 7:** Generated waveforms with lowest accuracy.

Waveform family	Waveform index	*mse* (normalized)	*mse* (original)
2^nd^	184	3.3616x10^-4^	1.4057x10^-2^
3^rd^	57	3.0750x10^-4^	1.3853x10^-2^

On the other hand, the minimum *mse* (highest accuracy) occurs for waveform 131 (2.0054x10^-5^ for the normalized version and 8.3857x10^-4^ for original version) in the 2^nd^ family and for waveform 192 (3.1278x10^-6^ for the normalized version and 1.4089x10^-4^ for the original version) in the 3^rd^ family.

**Fig 17:**
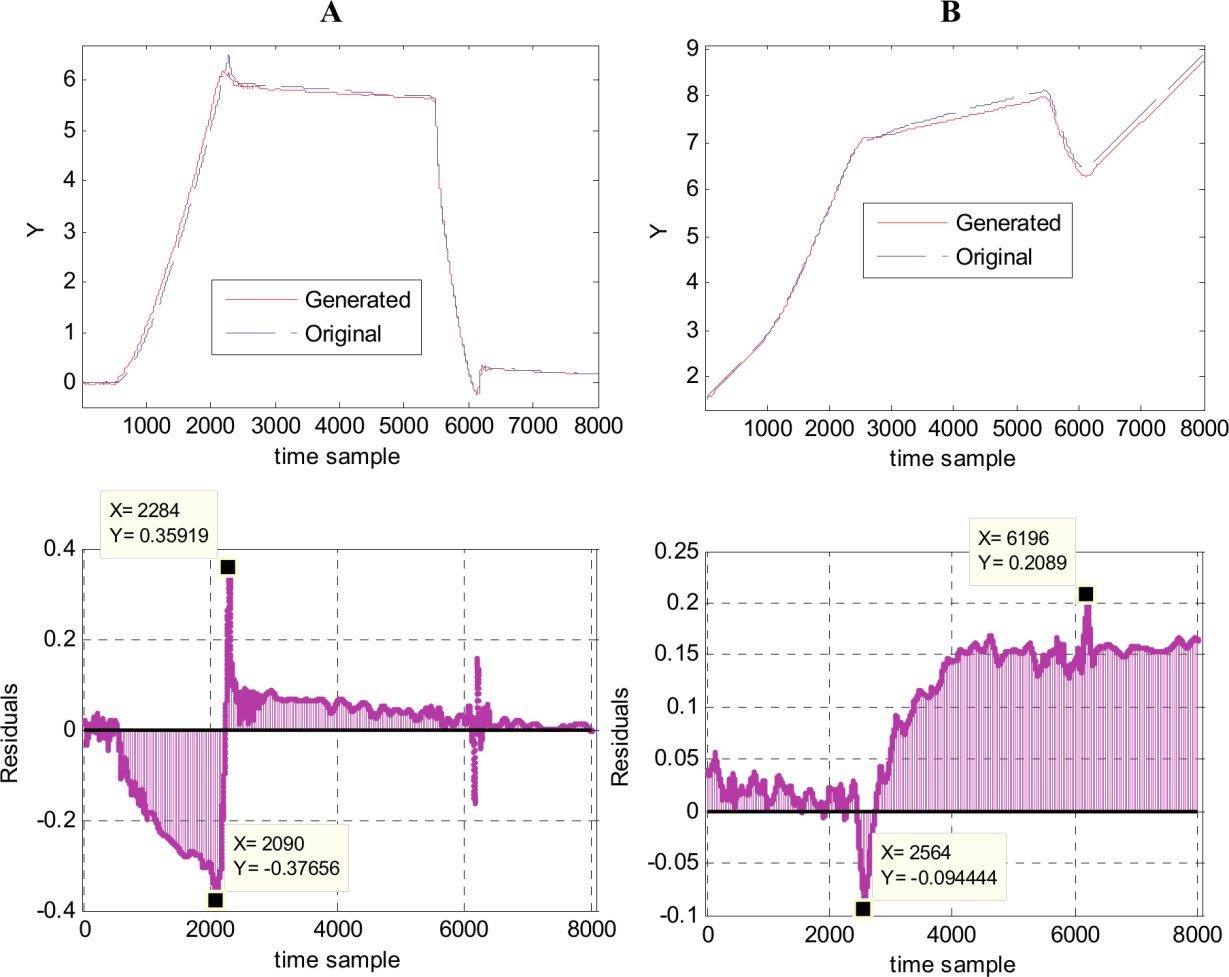
Comparison between the generated and original waveforms with highest *mse* (lowest accuracy). (A) 2^nd^ family–waveform 184 (generated vs. original waveform–top; residuals—bottom). (B) 3^rd^ family–waveform 57 (generated vs. original waveform–top; residuals—bottom).

Another perspective for the metamodel accuracy can be offered by the images in Fig 17 that presents a direct comparison between the original and predicted waveforms, for the lowest accuracy generated waveform in each family. For both families, even in the case of lowest accuracy, the generated waveforms are close enough to their original counterparts.

For the 2^nd^ family some differences between the original and predicted waveforms can be observed on the positive slope, overshooting, and almost constant high value regions (Fig 17A top). The residuals (Fig 17A bottom) are negative in the positive slope region with a maximum magnitude of 0.37656 for time sample 2090, and positive in the rest, with a maximum magnitude of 0.35919 at time sample 2284. The metamodel for this 2^nd^ waveform family is an accurate one, possessing a good generalization capability.

For the 3^rd^ family some differences between the original and predicted waveforms appear mostly in the second and third regions, starting from sample 2450 (Fig 17B top). The residuals are mainly positive, with a maximum magnitude of 0.2089 for time sample 6196, where the sample value should be 6.5320 and it is 6.3231. For this 3^rd^ family, the resulting errors are mainly due to the fact that it presents an important dispersion from one waveform to another (see also Fig 15B).

**Fig 18:**
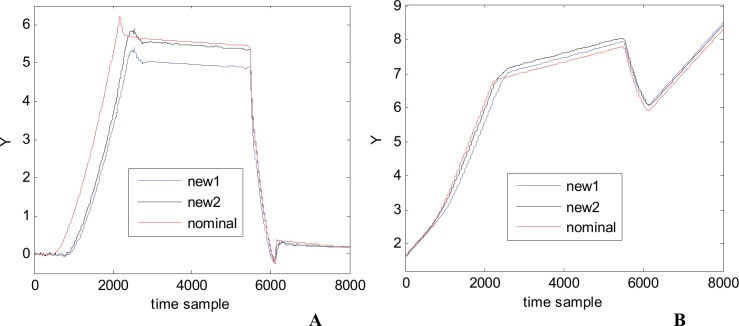
Two new generated waveforms and the nominal waveform: (A) 2^nd^ waveform family. (B) 3^rd^ waveform family.

Fig 18 presents two completely new waveforms for two new parameters combination, different from the ones used to build and test the metamodel. For both 2^nd^ and 3^rd^ families our metamodels generate trusted waveform as they share the same characteristics with the nominal waveforms and the other waveforms in each family (see also Fig 15).

## Discussion

A synthesis of the accuracy of our metamodels, measured by the mean squared error (*mse*) between original and generated waveforms, in the normalized version for our three waveform families is illustrated in Table 8. Because the original values of the waveforms vary a lot in the three families, the *mse* presented in Table 8 are the ones corresponding to the unuity-based normalized version. As a whole, the accuracy is similar for all three families, both mean and maximum values of *mse* having the same magnitude order (10^−5^ for mean value, respectively 10^−4^ for the maximum value.). The minimum *mse* values are similar for the 1^st^ and 3^rd^ families (order of magnitude 10^−6^) and one magnitude order greater for the 2^nd^ family (10^−5^). According with the numerical values, it appears that the 2^nd^ family is a little bit more difficult to be modeled, presenting the largest *mse* (7.0648x10^-5^ mean value, 2.0054x10^-5^ minimum value, and 3.3616x10^-4^ maximum value). Anyhow, it results that all metamodels generate accurate results for all waveform families, which validates both the proposed metamodel structure and the development procedure.

**Table 8:** Synthesis of *mse* of the generated waveforms with the metamodels in all three waveform families.

Waveform family	Mean squared error (*mse*)		
	Mean value	Minimum value	Maximum value
1^st^ family	3.1837x10^-5^	3.0273x10^-6^	2.8418x10^-4^
2^nd^ family	7.0648x10^-5^	2.0054x10^-5^	3.3616x10^-4^
3^rd^ family	2.2924x10^-5^	3.1278x10^-6^	3.0750x10^-4^

From the point of view of computational effort it is important to mention the necessary hardware resources and the CPU time. The development of metamodels was realized on a general purpose computer (i5-4460 CPU @ 3.2GHz, 8GbRAM, 64-bit operating system). The time necessary for metamodel development (considering that the necessary numerical data–waveform families—is available) was maximum 18 min for the 1^st^ family. For the other two families, the time was slightly shorter (11 min and 30 s for 2^nd^ family and 14 min for the 3^rd^ family).

Considering the fact that the metamodel should be developed only once for every waveform family, a time of maximum 18 min is fully convenient, from the practical point of view. Indeed, if the number of available waveforms in the family increases, or if the number of time samples increases, the development time increases as well, but it remains acceptable, in terms of tens of minutes.

To generate a new waveform for a new parameter combination, our metamodel does the job in less than 1 s (0.947 s). This means that even if we want to perform extensive waveform generations, the necessary time to generate them remains a practical one, for example to generate 1000 new waveforms, we need less than 1000 s, meaning less than 17 min.

The metamodels presented here make for cost-effective, but reliable replacements of complex systems (ECU) that empower the design team to perform extensive analyses of system response, under a theoretically infinite number of possible combinations of input parameters. This is very useful, because multiple simulations of a detailed model of the system lead to a very long simulation time, which is usually prohibitive.

Since the running time of our metamodel takes less than 1s, it can be used for efficient generation of not-yet simulated output waveforms, for any possible combination of dependent parameters, offering the possibility to cover the entire design space. If the designer is able to do that, a wide range of possibilities become achievable, such as: all design corners can be explored, possible worst-case situations can be investigated, extreme values of waveforms can be discovered, sensitivity analyses can be performed (the influence of each parameter on the output waveform), and so on.

Now, if the designer possesses such a promising metamodel, he/she faces a new problem: what is the best approach to generate new parameter combinations for a well-controlled, systematic data collection, or, in other words, the design of experiments, DoE. There are a lot of methods to be used, for example: full factorial design, fractional factorial design, response surface design, D-optimal design, latin hypercube design, quasi random design ([36–37]) or sequential optimal space [38]. Two important DoE concepts, i.e. blocking and replications, can be used in order to improve the analysis of the electronic system performance in the case of variations of both factors and measurements [39].

Our metamodels satisfy the main required key points:

- accuracy–our models are capable of generating the system response over the design space, the maximum *mse* being 3.3616x10^-4^ for the 2^nd^ family (see Table 8);
- efficiency–the computational effort required for constructing the metamodel is a fully affordable one: a maximum of 18 min for the metamodel for 1^st^ family on a general purpose computer (i5-4460 CPU @ 3.2GHz, 8GbRAM, 64-bit operating system);
- simplicity–the user can easily run the metamodel in less than 1 s, on a general purpose computer: the user should only provide the combination of input parameters and the metamodel will generate the output waveform in a vector (sample by sample) and as a plot.

If, in some special cases, a higher exactitude of generated waveform is necessary, it is possible to achieve this using the proposed method. There are two main directions that can be followed. The first one consists in increasing the number (or ratio) of selected coefficients for the wavelet transform in the GA optimization, to capture more information about the waveform family. Of course this will lead to a certain increase of the metamodel’s complexity, by increasing the number of ANN neurons in the output level. This may also require a larger data training set. In fact, this is the second course of action, to increase the available data for metamodel development, especially for the ANN learning process. In this case, the developer should also consider the necessary resources for obtaining more numerical data by performing costly experiments and/or measurements.

## Conclusions

This paper describes a method to develop metamodels for the efficient generation of not-yet simulated waveforms as a function of different combinations of input parameters. The method is based on three main pillars: a GA optimization to find the optimal wavelet transform and to identify the relevant coefficients, an ANN that produces the relevant coefficients of the wavelet transform, and the wavelet decomposition and reconstruction transforms. Our method was applied to develop three metamodels that generate the waveforms in three points of an electronic control unit in the automotive industry. The accuracy of metamodels, by comparing the generated waveforms with original waveforms in the data set, proves to be a good one, the *mse* being around 3x10^-4^. Using the metamodel is very simple; the user only has to provide the current combination of input parameters and he/she will receive the corresponding waveform as a plot and as a sample-by-sample vector in less that 1s on a general purpose computer. The computational effort to develop the metamodel (once we have the necessary data set, a priori obtained by simulations and/or experiments) is a very convenient one. In our experiments, using a general purpose computer, a data set with 200 waveforms for 200 different combinations of 10 input parameters, the time spent for metamodel development is maximum 18 min.

The metamodels present all the necessary key features to be further used in system analyses and design, through extensive waveform generation, so that the entire design space is covered, by applying any possible input parameters combination.
